# Local detection of pH-induced disaggregation of biocompatible micelles by fluorescence switch ON†

**DOI:** 10.1039/d2sc00304j

**Published:** 2022-03-10

**Authors:** Giulia Battistelli, Maria Proetto, Alexandra Mavridi-Printezi, Matteo Calvaresi, Alberto Danielli, Paolo Emidio Constantini, Claudia Battistella, Nathan C. Gianneschi, Marco Montalti

**Affiliations:** Department of Chemistry “Giacomo Ciamician” Via Selmi 2 Bologna 40126 Italy marco.montalti2@unibo.it; Department of Chemistry Northwestern University Evanston IL 60208 USA; Department of Materials Science and Engineering Northwestern University Evanston IL 60208 USA; Department of Biomedical Engineering Northwestern University Evanston IL 60208 USA; FaBiT, Department of Pharmacy & Biotechnology, University of Bologna via Selmi 3 40126 Bologna Italy

## Abstract

Fluorogenic nanoparticles (NPs) able to sense different physiological environments and respond with disaggregation and fluorescence switching OFF/ON are powerful tools in nanomedicine as they can combine diagnostics with therapeutic action. pH-responsive NPs are particularly interesting as they can differentiate cancer tissues from healthy ones, they can drive selective intracellular drug release and they can act as pH biosensors. Controlled polymerization techniques are the basis of such materials as they provide solid routes towards the synthesis of pH-responsive block copolymers that are able to assemble/disassemble following protonation/deprotonation. Ring opening metathesis polymerization (ROMP), in particular, has been recently exploited for the development of experimental nanomedicines owing to the efficient direct polymerization of both natural and synthetic functionalities. Here, we capitalize on these features and provide synthetic routes for the design of pH-responsive fluorogenic micelles *via* the assembly of ROMP block-copolymers. While detailed photophysical characterization validates the pH response, a proof of concept experiment in a model cancer cell line confirmed the activity of the biocompatible micelles in relevant biological environments, therefore pointing out the potential of this approach in the development of novel nano-theranostic agents.

## Abstract 2

pH-responsive micelles disassembly, upon acidification during lysosomal uptake, leads to fluorescence switch ON. These nanoparticles are promising candidates for the design of novel stimuli-responsive drug delivery systems.

## Graphical abstract

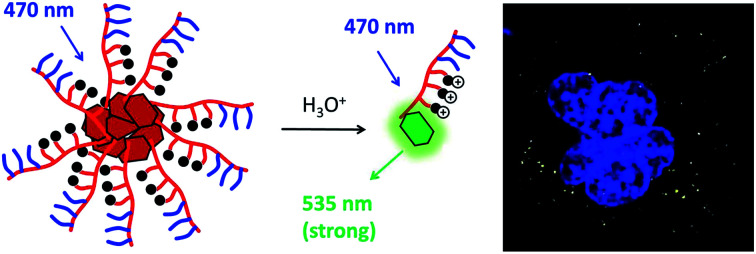

## 1 Introduction

Nanomedicine exploits defined nanostructures for the development of new systems with improved selectivity and efficiency with respect to existing diagnostic and therapeutic methods.^1,2^ As an example, nanoparticles (NPs) are presently used for the delivery of mRNA in many anti-COVID-19 vaccines.^3^ In this framework, “smart NPs”, able to respond to either internal or external stimuli, have offered great advantages in terms of versatility, controllability and specificity.^4–9^ Amphiphilic block co-polymers have emerged as one of the most promising approaches for the design of these stimuli-responsive nano-theranostic agents, as they can assemble into NPs of controlled size and surface properties.^10^ Additionally, the stability of these self-assembled NPs can be controlled^11^ by tuning parameters like the block co-polymer composition,^12,13^ the chain-length or the polymerization method.^14^ As a consequence, stimuli-induced changes in the block co-polymer composition can be easily exploited to decrease NP stability inducing disassembly.

### 1.1 Stimuli-responsive NPs

To obtain stimuli-responsive NPs, the polymeric units should include structure specific blocks sensitive to a selected stimulus that can be exploited to trigger their conversion from stable to unstable and if possible their disaggregation.^15^ Control over the polymeric composition is, in this context, fundamental since the overall response, and hence NP disaggregation, arises from the individual response of the single polymeric unit that has to be the same in order to achieve a sharp effect. Between the different stimuli that can be exploited to induce NP disaggregation, pH decrease is particularly important in nanomedicine. Cancer tissues, for example, possess a particularly acidic environment, which can be exploited as the stimulus for the selective response of pH-sensitive NPs.^16^ Intracellular vesicles such as endosomes and lysosomes are known to undergo progressive acidification,^17^ a process which can be exploited to trigger NP disassembly and intracellular drug release. Nevertheless, although this pH-induced disassembly process of NPs is very important for therapeutic application, the actual visualization of the disaggregation of the NPs in the target tissue is not trivial and it can be achieved by a tailored functionalization of the polymers with specific fluorescent molecules through which fluorescence switching OFF/ON upon NP disassembly can be achieved. Fluorescence imaging is, in fact, a powerful tool for the visualization of biological events thanks to its high specificity and sensitivity,^18^ while various organic fluorescent molecules have been proposed for the development of diagnostic and sensing platforms.^19^ For the design of fluorogenic NPs, although non-covalent integration of the emissive component in the system is synthetically easy and convenient,^20^ the covalent binding of the fluorophore to the NP guarantees higher specificity preventing fluorophore leaking and unspecific responses.

### 1.2 Disaggregation induced emission (DIE)

The strategy we propose here to achieve a fluorogenic response to disassembly is based on disaggregation induced emission (DIE).^21^ This approach presents, for the visualization of NP disassembly, an advantage with respect to alternative aggregation induced emission (AIE) since it offers the possibility to observe a switch ON of fluorescence upon disaggregation instead of fluorescence quenching, which would be less specific.^22,23^ Perylene diimides (PDIs) constitute an important class of organic dyes that present ideal features for the design of systems whose response is based on the fluorescence signal switch ON or OFF upon aggregation–disaggregation processes.^24–28^ In fact, PDI dyes, which are very strongly fluorescent, exhibit a very low fluorescence quantum yield in the aggregated form.^29^ As one of the main prerequisites for bio-application is the high biocompatibility of the probe, organic assemblies, consisting of a fluorescent core made by an organic emitter and a shell made by biocompatible/biodegradable polymers, are very promising candidates. Here, we describe the preparation and characterization and we also demonstrate the bio-application of NP (micelles) that: (i) result from the self-assembly of block-polymers with a well-defined composition and bear a pH-responsive block, (ii) are stable at pH 7.4, but disaggregate upon moderate acidification (pH 5.0), (iii) their disassembly can be detected since it is associated to a clear switch ON of the fluorescence (DIE), (iv) are different from similar previously reported systems,^23,30^ and whose fluorescence change is considerable and is demonstrated in a significant biological model. The micelle constituting units are amphiphilic block co-polymers terminating with a single PDI unit prepared by exploiting ring opening metathesis polymerization (ROMP) of functionalized norbornene derivatives. ROMP was the synthetic approach of choice due to the high control that it endows to the system, leading to uniform populations.^31,32^ Moreover, ROMP recently emerged as a powerful polymerization technique in nanomedicine as both natural motifs and targeting moieties or therapeutics can be easily polymerized.^33–37^ Therefore, in this study we aimed to exploit such a polymerization technique to prepare micellar NPs that are able to respond to relevant physiological environments with fluorescence “switch ON”.

### 1.3 pH-responsive micelles

**Scheme 1:**
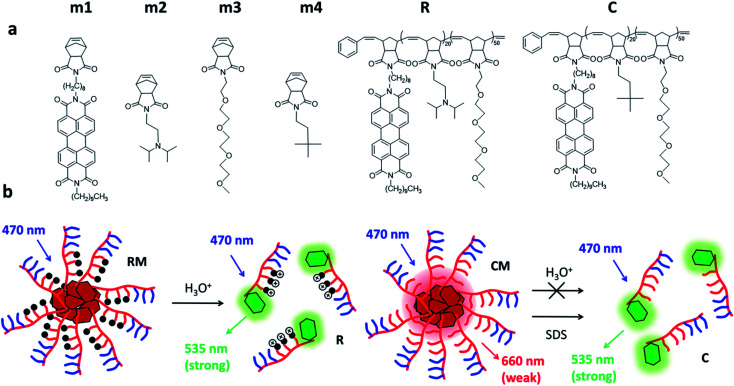
(a) Chemical structures of the monomers (m1–m4) and the amphiphilic block copolymers R and C. Both R and C are functionalized with a fluorescent perylene derivative which can be excited at 470 nm. (b) Polymers C and R aggregate into micellar assemblies. In the case of R micelles (RM), acidification triggers the disaggregation of the micelles leading to fluorescence “switch ON”. In contrast, pH variation does not cause the disassembly of C micelles (CM), which can be disaggregated in the presence of a detergent (SDS).

Herein, we constructed amphiphilic block copolymers capable of being formulated into NPs, utilizing three different norbornene monomers selected from the four m1, m2, m3 and m4 shown in Scheme 1a. More in detail, the first polymer R is constituted by a PEGylated hydrophilic block (m3 block) and a tertiary amine pH-responsive hydrophobic block (m2 block) efficiently functionalized by a fluorescent perylene derivative (m1). The polymer’s intrinsic tendency to self-assemble was exploited to obtain spherical micelles (RM), where the variation of the pH from 7.4 to 5.0 led to the fluorescence “switch ON”. As shown in Scheme 1b, emission is triggered due to the protonation of the amine of the hydrophobic block m2, causing a structural change in the aggregation properties of the amphiphiles and the PDI derivative. A second polymer C constituted by a PEGylated hydrophilic block (m3 block) and a pH insensitive hydrophobic block (m4 block) efficiently functionalized by a fluorescent perylene derivative (m1) was prepared as the control. The copolymer C was assembled into micelle CM which did not undergo disassembly in acidic conditions, but could be disaggregated in the presence of a surfactant such as sodium dodecyl sulphate (SDS).

This work provides a careful optical characterization of the obtained micelles at different pH levels of interest and demonstrates a simple approach to generate novel responsive nanomaterials for the development of nano-theranostic and drug delivery tools. Herein, as a proof of concept, *in vitro* experiments in a model cancer cell line are reported to prove the biocompatibility and cellular internalization and to validate the effective responsiveness of RM to relevant physiological environments.

## 2 Results and discussion

Fluorogenic micelles were prepared *via* the self-assembly of polymeric amphiphilic units R and C (synthesized by ROMP polymerization as described in the ESI†) as shown in Scheme 1a. In particular, polymer R contains a section of amino terminated units (p*K*_a_ = 6.9) that switch from being hydrophobic to hydrophilic upon protonation. Both polymers R and C were characterized by size exclusion chromatography (SEC) showing a *M*_W_ of 21 060 g mol^−1^ (*Đ* = 1.069) and 18 070 g mol^−1^ (*Đ* = 1.013) respectively (Table S1†).

**Fig. 1:**
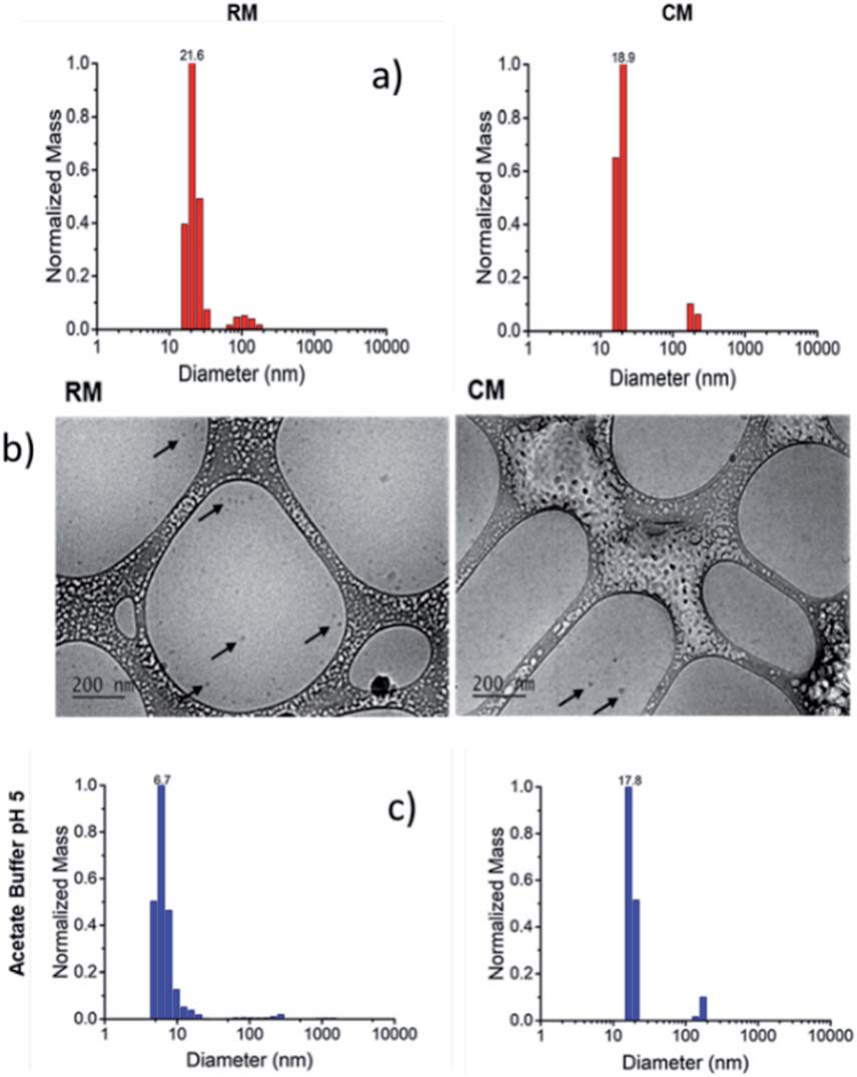
(a) Size distribution of RM and CM at pH 7.4 obtained by DLS. (b) Cryo-TEM images of RM and CM. (c) Size distribution, obtained by DLS, of RM and CM after 48 h dialysis against a buffered solution at pH 5.0.

For the preparation of the micelles, the polymers were dissolved in dimethylformamide (DMF) and the solvent was slowly replaced by dialysis into PBS for 72 h. The micelles resulting from the assembly of R and C will be referred to as RM and CM respectively (Scheme 1b) and their formation was demonstrated both by dynamic light scattering (DLS) and by cryo-TEM as shown in Fig. 1. Both types of micelles showed a hydrodynamic diameter *d* ∼20 nm.^38,39^

We would like to emphasize that RM contains a block of the amino terminated monomer m2 which is mostly unprotonated and hydrophobic at physiological pH (7.4), but undergoes protonation at acidic pH becoming hydrophilic. Hence, RM stability is expected to be strongly pH-dependent and to decrease at low pH leading to disassembly triggered by the formation of a hydrophilic protonated R polymer (according to Scheme 1b). In contrast, micelles CM do not contain protonatable sites and the m4 block remains hydrophobic independent of the pH. As a consequence, the stability of CM is not expected to be pH-dependent.

As shown in Scheme 1a, both the polymeric chains R and C possess a starting monomeric unit m1, which contains a perylene diimide (PDI) chromophore. PDI derivatives are known to give strong π–π interactions that lead to the formation of aggregates with altered photophysical properties with respect to the starting monomer. In particular, the formation of H aggregates leads to a distortion of the absorption band and to considerable quenching of the fluorescence.^29^ Hence, both the formation of the micelles and possible disaggregation processes can be studied by photophysical measurements.

### 2.1 Photophysical properties

**Fig. 2:**
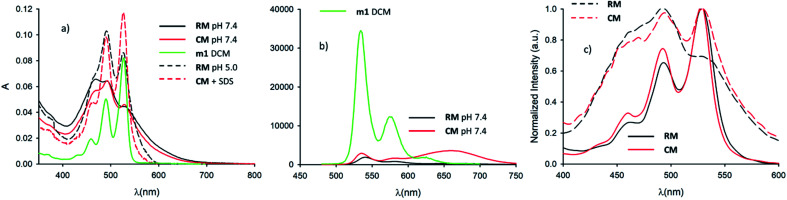
(a) Absorption spectrum of m1 in CH_2_Cl_2_ (*c* = 10^−6^ M) (green line). Absorption spectra of micelle RM at pH 7.4 (continuous black line) and 5.0 (dashed black line). Absorption spectra of micelle CM at pH 7.4 in the absence (continuous red line) and in the presence (dashed red lines) of SDS. (b) Fluorescence spectra of m1 in CH_2_Cl_2_ (*c* = 10^−6^ M) upon 470 nm excitation (green line). Fluorescence spectrum of CM upon 470 nm excitation at pH 7.4 (red line). Fluorescence spectrum of RM upon 470 nm excitation at pH 7.4 (red line). (c) Excitation spectra of RM and CM at *λ*_em_ = 580 nm (continuous black and red lines respectively) at pH 7.4. Excitation spectra of RM and CM at *λ*_em_ = 660 nm (dashed black and red lines respectively) at pH 7.4.

In order to understand the effect of micelle formation on the photophysical properties of the PDI chromophore, we first investigated the absorption and fluorescence properties of the reference precursor m1 in dichloromethane (CH_2_Cl_2_, DCM), a solvent where PDI molecules aggregate very poorly. The absorption spectrum of m1 in CH_2_Cl_2_ (1 × 10^−6^ M), shown in Fig. 2a, clearly shows the typical vibration structure of the lowest energy absorption band of the PDI monomer with a maximum at 526 nm (*ε* ∼80 000 M^−1^ cm^−1^).^26,27^ A similar vibrational structure is observed for the fluorescence band upon excitation at 470 nm as shown in Fig. 2b. This is the expected emission of the monomer, with a maximum at 535 nm and a fluorescence quantum yield *Φ* = 90%.^29^ The attribution of the fluorescence to the monomeric species was confirmed by the excitation spectrum at 575 nm (not reported) that matched the absorption spectrum of the monomer shown in Fig. 2. The excited state lifetime was measured by time correlated single photon counting (TCSPC): the kinetic trace was fitted with a mono-exponential decay giving a lifetime *τ* = 4.6 ns (see 1.3.1 of ESI†).

**Fig. 3:**
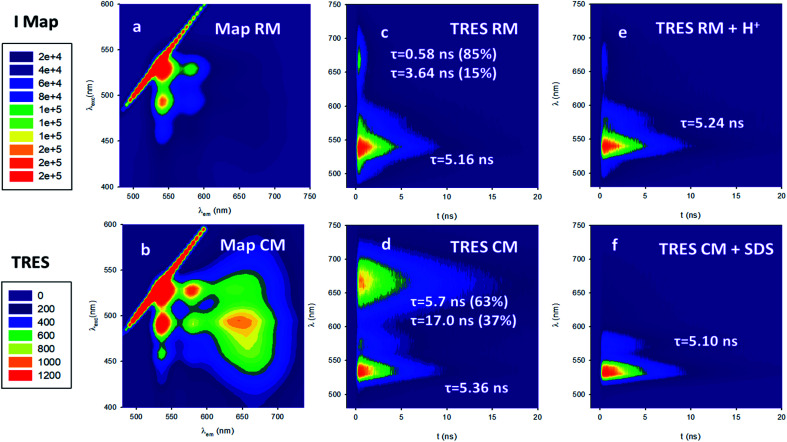
(a) Fluorescence map of RM. (b) Fluorescence map of CM. (c) Time resolved emission spectra (TRES) of RM with a mono-exponential lifetime of *τ* = 5.16 ns for the green emission. The lifetimes also at 660 nm and 540 nm are found to be 0.58 ns and 3.64 ns respectively. (d) TRES of CM with a mono-exponential lifetime of *τ* = 5.36 ns for the green emission. The lifetimes also at 660 nm and 54 0 nm are found to be 5.7 ns and 17.0 ns respectively. (e) TRES of the disaggregated RM at pH = 5.0 fitted with a mono-exponential lifetime of *τ* = 5.24 ns (f) TRES of CM upon disaggregation with SDS fitted with a mono-exponential lifetime of *τ* = 5.10 ns.

**Fig. 4:**
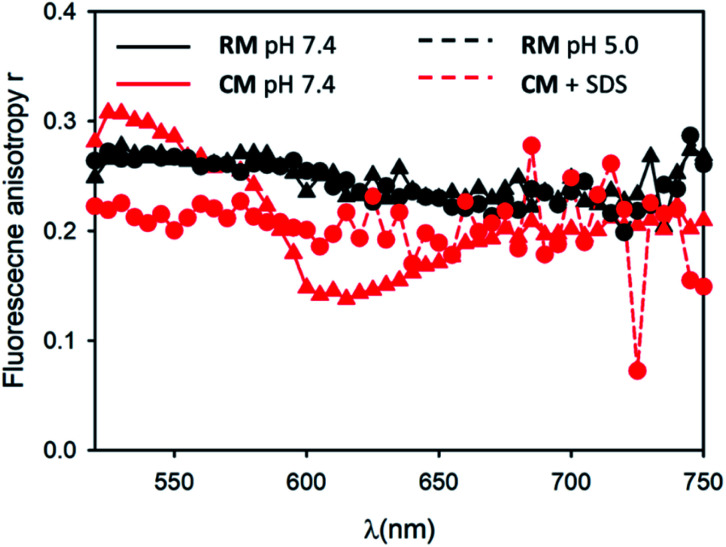
Fluorescence anisotropy spectra of RM at pH 7.4 (black circles) and pH 5.0 (black triangles). Fluorescence anisotropy spectra of CM in the absence (red circles) and in the presence of SDS (red triangles).

Following this, we investigated the photophysical properties of the micelles RM and CM in PBS at pH 7.4. The absorption spectra of the micelles RM and CM (in a concentration of the polymeric components R and C of 1.5 × 10^−6^ M) are shown in Fig. 2a and they clearly show a distortion of the absorption spectrum that results from the π–π interaction of the PDI units at the core of the micelles. In both cases, the absorption maximum is shifted to lower wavelengths with respect to the monomer (*λ*_max_ = 469 nm and *λ*_max_ = 492 nm for RM and CM respectively) and it is consistent with the formation of H-aggregates in the cores of the micelles.^29^ This conclusion is also supported by the quenching of fluorescence of the micelles with respect to the monomer. The fluorescence spectra, upon excitation at 470 nm, are shown in Fig. 2b. For the CM micelles, two fluorescence bands are present at 536 and 661 nm, the latter being more intense than the former. For RM, in contrast, the main fluorescence band, upon 470 nm excitation, is at 541 nm and only a weak emission can be observed at 668 nm. While the bands at 536 and 541 nm (green fluorescence) present the typical energy and structure of the PDI isolated chromophore, the bands at 661 and 668 nm (red fluorescence) correspond to emissions that have been attributed, for similar systems, to the presence of aggregates, and they arise from delocalized excited states with lower energy and unstructured fluorescence.^26^ It is noteworthy that for both RM and CM the average quantum yield was quite low being 5% and 12% respectively (see 1.3.2 of the ESI†). The origin of high energy green fluorescence was confirmed using the excitation spectra (*λ*_em_ = 580 nm), shown in Fig. 2c, that presented the typical structured band of the monomer. For a more detailed investigation, the fluorescence maps of RM and CM were acquired and are shown in Fig. 3 (a and b respectively). These contour maps represent the fluorescence intensity as a function of the excitation and emission wavelength and they allow us to correlate the excitation and emission spectra of the different species present in a sample. The emission map of RM clearly shows the dominant presence of the green structured emission independent of the excitation wavelength while, in the case of CM, two different peaks can be clearly identified, corresponding to the green and red emissions. In addition, TCSCP was used to acquire the time resolved emission spectra (TRES) of RM and CM as shown in Fig. 3 (c and d respectively). TRES maps allow us to follow the kinetics of excited state deactivation at different wavelengths. It is interesting to note that both in the case of RM and CM the green emission decays can be fitted with a mono-exponential model with lifetimes (5.16 and 5.36 ns for RM and CM respectively), which are comparable to the one measured for the non-aggregated m1 in CH_2_Cl_2_. These results suggest that the green emission is indeed originated from a fraction of the polymeric chains R and C which is not assembled into the micelles, and hence in the micellar solution the structured emission band is due to a minor fraction of the unquenched, free polymeric moieties. Furthermore, the presence of large disassembled highly emitting molecules was confirmed by anisotropy fluorescence measurements (shown in Fig. 4). This technique provides information about the kinetics of the reorientation processes occurring between the excitation and the emission of a fluorophore and, in particular, about the rotation rate. Fluorescence anisotropy in the 530–540 nm range was as high as *r* = 0.27 and *r* = 0.28 for RM and CM respectively, suggesting that the rotation of the PDI fluorophore is slow since it is bound to a high molecular weight polymer.

Having clarified that the residual green emission was due to un-associated polymeric units, it was possible to use the fluorescence quantum yield of this emission to quantify the fraction of free polymeric chains that was about 6% in the case of RM and about 10% in the case of CM (see 1.3.3 of the ESI†). As previously mentioned, in the case of CM a second broad and intense fluorescence band in the red region was detected. This fluorescence is typical of PDI aggregates and, hence, it is originated by the CM micelles themselves. This is also demonstrated by the excitation spectrum at *λ*_em_ = 660 nm (shown in Fig. 2c) that presents the profile of the PDI aggregates. We would also like to stress that, unlike what is observed for the dynamic formation of an excimer where a delay time for the formation of an excimer species is detected (since it requires the encounter of an excited and a ground state chromophore), in the case of CM the TRES map shown in Fig. 3d reveals that the formation of the red emitting species is fast (<100 ps). In particular, this formation is much faster than monomer excited state decay, demonstrating that the two processes (monomer excited state decay and exciplex formation) are not correlated and hence confirming that the two emissions can be attributed to two different species. As shown in Fig. 3d the decay of the red fluorescence of CM could be fitted with a biexponential model to give two lifetimes as long as 5.7 and 17.0 ns. In contrast, in the case of RM, the red emission is not only much weaker than the green one, but as shown in Fig. 3c, it is also much short-lived than that of CM. In fact, in this case, it could be fitted with a bi-exponential model giving two lifetimes as short as 0.58 and 3.64 ns.

This behaviour can be easily explained by the presence of unprotonated m2 units in the micelles. In fact, it is well known that amino derivatives are able to quench excited PDI molecules *via* an electron transfer mechanism. Therefore, as shown in Scheme 1b, we can assume that the unprotonated amino group in the micelles indeed produces fast deactivation of the PDI excited state leading to complete quenching of the fluorescence of the micelles. In summary, we can conclude that at pH 7.4: (i) both R and C are, to a large extent (more than 90% in molar fraction), assembled into micelles; (ii) a residual green fluorescence can be observed in both cases due to the presence of a minor fraction of disassembled polymers; (iii) micelles CM show a broad fluorescence band in the red region with a long excited state lifetime; (iv) micelles RM show only a weak emission in the red region with a much shorter lifetime with respect to CM. This is due to quenching of the emission due to electron transfer processes involving unprotonated amino groups present in the micelles.

### 2.2 Disaggregation experiments

**Fig. 5:**
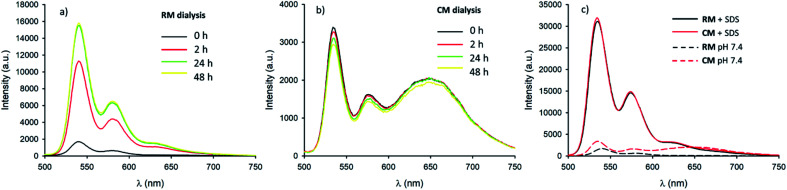
(a) Fluorescence spectra (*λ*_exc_ = 470 nm) of RM in PBS pH 7.4 before (black line), and after dialysis against acetate buffer pH 5.0 (red line 2 h, green line 24 h and yellow line 48 h). (b) Fluorescence spectra (*λ*_exc_ = 470 nm) of CM in PBS pH 7.4 before (black line), and after dialysis against acetate buffer pH 5.0 (red line 2 h, green line 24 h and yellow line 48 h). (c) Fluorescence spectra (*λ*_exc_ = 470 nm) of RM and CM before (black and red dashed lines respectively) and after (black and red continuous lines respectively) addition of a 10% SDS solution.

Micelles RM were designed to present pH-responsive disaggregation attributed to the presence of the alkyl–amine moieties that are protonated at a pH below neutral and similar to the physiological pH (p*K*_a_ = 6.9). In order to check this pH response, two different experiments were performed: (i) titration with an HCl solution to quickly decrease the pH and validate the system (Fig. S6 and S7†), and (ii) dialysis against an acetate buffer solution at pH = 5.0, to better mimic the pH found in physiological environments. In the latter case, acidification of RM was achieved *via* a dialysis experiment using a membrane with low permeability (cutoff = 3500 kDa) in order to avoid the out-diffusion of both micelles and polymeric units. A solution of RM in PBS was dialysed against acetate buffer at pH 5.0. In Fig. 5a the fluorescence spectra of RM before and after dialysis (2 h, 24 h and 48 h) are shown demonstrating that the pH decrease produces an increase in the fluorescence intensity by about 9 times after 48 h dialysis. We would like to stress that much of the fluorescence increase in the RM sample occurs within the first 2 hours of dialysis against acidic acetate buffer. In contrast, when the same experiment was performed on the control sample, CM, as shown in Fig. 5b, almost no change in the fluorescence spectrum was observed upon decreasing the pH from 7.4 to 5.0 even after 48 h dialysis.

In order to understand in detail the pH-responsive fluorescence of RM, we performed a comprehensive photophysical characterization of the systems at pH = 5.0. In particular, as shown in Fig. 2a, the absorption spectrum of RM greatly changed by changing the pH from 7.4 to 5.0 exhibiting the typical features of non-aggregated PDI in the acidic solution.^24,26^ The disaggregation was confirmed by the average fluorescence quantum yield of the RM sample, which increased to 46% upon decreasing the pH from 7.4 to 5.0. The TRES map of RM at pH = 5.0 is shown in Fig. 3e. It is worth mentioning that it was possible to fit the decay of the green fluorescence with a mono-exponential model demonstrating that disaggregation did not lead to any significant change in the fluorescence excited state lifetime, that was *τ* = 5.24 ns at pH 5.0. This result clearly shows that acidification indeed produced an increase in the fraction of free R molecules, as a result of the disaggregation of the micelles due to the protonation of the amino groups. Additionally, as shown in Fig. 4, fluorescence anisotropy of RM did not change by decreasing the pH from 7.4 to 5.0 demonstrating that at both pH levels, the fluorescent species are the same and are the free polymer chains.

For the CM system, the pH decrease from 7.4 to 5.0 had no effect on aggregation due to the absence of easily protonatable sites in C. Nevertheless, as a control experiment, micelles CM were treated with a surfactant (SDS) which is known to solubilize the polymers leading to micelle disaggregation. As shown in Fig. 2a, the absorption spectrum of CM completely changed after the addition of SDS presenting the typical structure of non-aggregated PDI. Correspondingly, as shown in Fig. 5c, the red emission attributed to the micelles completely disappeared while a strong increase of the green fluorescence of polymer C could be detected. In the same Fig. 5c, a very similar behaviour can be observed for RM. The final fluorescence quantum yield of CM after the addition of SDS was as high as 87% and so was compatible with complete disaggregation of the micelles. The TRES map of CM in the presence of SDS is shown in Fig. 3f and it confirms the complete disappearance of the red emission band.

Moreover, the green fluorescence band could be fitted with a single mono-exponential decay with a lifetime *τ* = 5.10 ns very similar to the one measured in the absence of SDS. As far as fluorescence anisotropy is concerned, as shown in Fig. 4, the addition of SDS leads to a decrease of the anisotropy of CM that in any case remains as high as *r* = 0.22. This result is compatible with the incorporation of more than just one C molecule in the SDS micelles and a partial depolarization of fluorescence because of the homo-energy transfer between the PDI units. The disaggregation of RM micelles in this acidic environment (pH = 5.0) was also investigated by dynamic light scattering (DLS) experiments, as well as by TEM (Fig. 1, S8 and S9†). In this context, we would like to stress that neither DLS nor TEM allowed us to identify clear individual R or C molecules. Nevertheless, in the case of DLS, disassembly of the micelles is expected to produce a decrease in the scattering intensity detected by the instrument as the single disaggregated molecular units exhibit poor scattering. Indeed, in the case of RM, acidification of the solution at pH 5.0 upon dialysis led to a decrease in the scattering intensity of about two orders of magnitude verifying the disaggregation of most of the RM micelles to poorly scattering molecular units. However, as shown in Fig. 1c, this corresponds to only a minor decrease in the hydrodynamic diameter related to the minor fraction of the still aggregated micelles, while a minor fraction of the larger aggregates is observed both before and after the pH change. Additionally, the residual fraction of undissociated RM could also be detected in TEM at acidic pH, which revealed the presence of residual particles after the acidification of RM (Fig. S9†).

A similar result was also observed in the case of acidification of RM by using HCl, where a decrease of about two orders of magnitude of the scattering intensity was noted without a substantial change in the measured hydrodynamic diameter of the few remaining highly scattering micelles (Fig. S7†). In contrast, no changes in the scattering intensity, in the hydrodynamic diameter and in the TEM images were detected upon acidification of CM with both acetate buffer and HCl, therefore confirming their stability after pH reduction (Fig. S7–S10†).

### 2.3 Cell culture experiments

Having demonstrated the disaggregation of RM upon acidification from pH 7.4 to pH 5.0 in a buffered solution, we then tested the ability of these micelles to respond to a physiological pH decrease in living cells. We would like to stress that although another pH-responsive PDI-based fluorogenic nanosystem has previously been reported, it did not show any disaggregation ability, and its application at the cellular level was not demonstrated.^40^ In particular the endo-lysosomal pathway of cells is characterized by a gradual pH decrease, a process that cells exploit for degradation of unnecessary cargo upon internalization. In this pathway, the drop in the pH ranges from 7.4 (extracellular) to 6.0 in the late endosomes and can reach values around 4.5 in lysosomal compartments.^17^ Self-assembled delivery systems capable of disassemblying upon such pH change are particularly interesting for pH-activated release of specific cargos. In general, verifying the actual disassembly of nano-vessels in response to acidification is not trivial; however regarding RM, on the basis of the previously discussed disaggregation, this process can be visualized in real-time by following the fluorescence response.

**Fig. 6:**
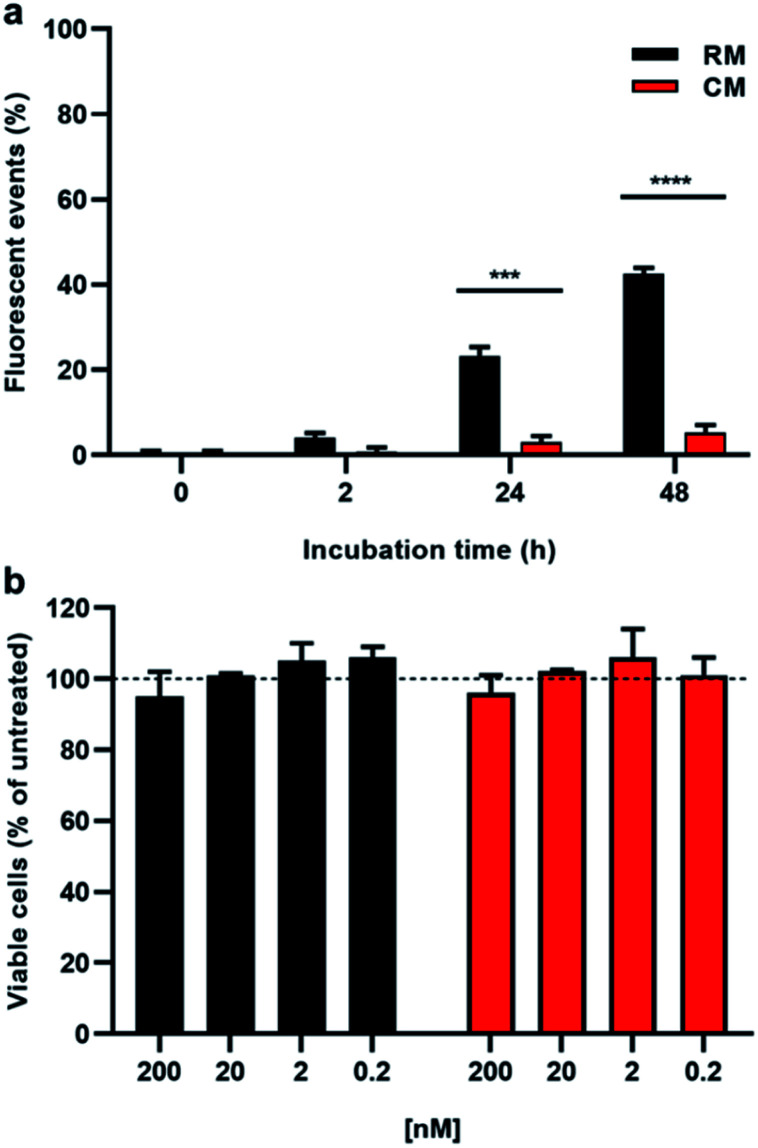
(a) Flow cytometry experiment reporting the fluorescence events after 2, 24 and 48 hour incubation of HeLa cells with 200 nM RM (black bars) and CM (red bars). (b) Cell viability as determined by MTT assay after 48 hour incubation of HeLa cells with increasing concentrations of RM (black bars) and CM (red bars) (200, 20, 2.0, and 0.2 nM). Asterisks indicate statistical significance calculated using the *t*-test (***p <0.001, ****p <0.0001).

HeLa cells, a model cancer cell line often used for cellular uptake studies,^41^ were incubated with either CM or RM and analysed by cytofluorimetry, a technique that allows the easy quantification of the fluorescence response in a cellular environment. As shown in Fig. 6a, cells treated with the responsive micelles RM show a gradual increase of the fluorescence that is, after 48 h incubation, almost one order of magnitude higher than the one observed in the presence of the control micelles CM. Moreover, in order to further test the biocompatibility of the probe, cell viability was assessed by MTT assay after up to 48 h incubation at increasing doses of both CM and RM. As shown in Fig. 6b and S12,† no significant reduced viability was observed in our experimental conditions upon either RM or CM incubation even at a concentration of 200 nM. This result demonstrates the biocompatibility of the developed micelles.

**Fig. 7:**
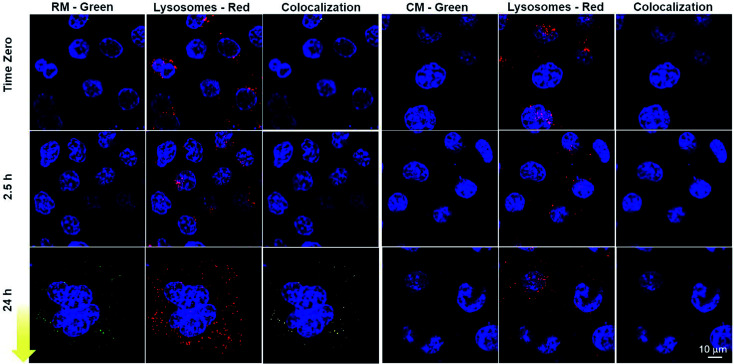
Confocal fluorescence microscopy of cells incubated with either RM or CM for 3 h. Cells were washed and the images were acquired immediately, as well as after 2.5 and 24 h. Disassembled micelles are visible in the green channel and lysosomal vesicles were stained with lysotracker red. Cell nuclei were stained with Hoechst (blue). The colocalization analysis of the green and the red channel is also illustrated: colocalization is represented as a white signal.

Although the cytofluorimetric experiment gave important quantitative information (Fig. 6a), which were in agreement with an increase of the RM fluorescence associated with endolysosomal acidification, confocal imaging experiments were needed in order to demonstrate (i) the actual internalization of RM by the cells through the endolysosomal pathway and (ii) the disaggregation of the micelles upon acidification. To this end, with a second experiment, cells were incubated with either RM or CM for three hours and then washed with PBS and observed using a confocal fluorescence microscope. In this way, the actual localization of fluorescence in the lysosomes and its temporal evolution (lysosome acidification is indeed a gradual process)^17^ could be visualized. In order to allow localization, cell lysosomes and nuclei were stained with lysotracker red and Hoechst respectively, and the fluorescence was monitored immediately after washing, as well as after 2.5 and 24 h incubation of the cells in micelle-free cell media (Fig. 7 and S13†). Fluorescence imaging revealed that 24 h incubation led to the appearance of punctuating green fluorescence dots in the case of RM. Such fluorescent signals were higher in number and intensity as compared to that of cells incubated with the control micelles (CM) and as compared to the initial time points for both RM and CM. In order to visualize the spatial overlap between the two fluorescent signals, the red of the lysotracker and green of the disaggregated RM and colocalization analysis of the green and the red channel for each incubation time (time zero, 2.5 h and 24 h) was performed using ImageJ software (Fig. 7 and Table S2†). As expected, in the case of CM, the disaggregation did not occur upon endocytosis even after 24 h incubation and therefore no overlay of the green and red channels was observed. In contrast, upon 24 h cell incubation with RM, when the channels were combined, a high number of overlapping pixels were represented as white dots.

Such results are compatible with the previously described micelle disassembly and therefore indicate that these pH-responsive micelles can be efficiently internalized by cells, that they can sense the different physiological pH values and that their disassembly, as a response to acidification, can be easily monitored owing to the fluorescence “switch ON”. This response to physiological environments is an interesting feature in nano-theranostics as it can be exploited to specifically target diseased tissues and control spatiotemporal drug release from nanocarriers as well as optical responses.^42^

## 3 Conclusions

pH-responsive fluorogenic NPs are of great interest in the field of nanomedicine as they can contribute to the development of both novel therapeutics and diagnostic tools. In this work, we demonstrated that ROMP polymerization, which is particularly interesting because it allows the direct polymerization of functional monomers, can be used to generate pH-responsive fluorogenic micelles. While such micelles are stable at physiological pH, they rapidly disassemble at pH 5 giving rise to fluorescence “switch ON”. Such a transition was further confirmed with *in vitro* experiments in a model cancer cell line, in which the drop in the pH along the endolysosomal pathway resulted in green fluorescence activation. This proof-of-concept work provides a powerful synthetic platform in which therapeutics of interest can be easily incorporated *via* direct polymerization using ROMP, allowing the development of novel nano-theranostic agents in which fluorescence activation is accompanied by drug release at the targeted site.

## Data availability

The data supporting the findings of this study are available within the article and in the ESI.†

## Author contributions

GB, CB and MP synthesized and characterized the polymers under the supervision of NCG. AM-P, GB and MM performed the photophysical characterization of the system. AD, PEC, CB and MC performed the cellular experiments. CB and MM conceived the experiment and wrote the manuscript.

## Conflicts of interest

There are no conflicts to declare.

## Supplementary Material

SC-013-D2SC00304J-s001
