# Supplementary material for: Local detection of pH-induced disaggregation of biocompatible micelles by fluorescence switch ON
Source: Chem Sci. 2022 Mar 10;13(17):4884–92. doi: 10.1039/d2sc00304j (PMC9067588; doi:10.1039/d2sc00304j)
Supplement: SC-013-D2SC00304J-s001 [file SC-013-D2SC00304J-s001.pdf]

Electronic Supplementary Material (ESI) for Chemical Science.
This journal is © The Royal Society of Chemistry 2022

# Supporting Information

# Local detection of pH-induced disaggregation of biocompatible micelles by fluorescence switching-on

Giulia Battistelli, Maria Proetto, Alexandra Mavridi-Printezi, Matteo Calvaresi, Alberto Danielli, Paolo Emidio Costantini, Claudia Battistella,* Nathan Gianneschi, Marco Montalti*

## 1. Materials and Methods

### 1.1 Materials

All reagents, solvents and chemicals were purchased from Sigma-Aldrich and used without modification, unless otherwise stated. Polymerizations were performed in dry nitrogen atmosphere using dimethylformamide (DMF) as the solvent. Modified second generation Grubbs ruthenium initiator, (IMesH2)(C5H5N)2(Cl)2Ru=CHPh, was prepared as previously indicated. [1]

### 1.2 UV-Vis Spectroscopy

The experiments were carried out in air-equilibrated solutions at 25 °C. UV–Vis absorption spectra were recorded with a Perkin-Elmer Lambda 650 or Perkin-Elmer Lambda 45 spectrophotometer using quartz cells with path length of 1.0 cm or 2.5 mL macro PMMA or UV disposable cuvettes purchased from BRAND.

### 1.3 Fluorescence Spectroscopy

The fluorescence spectra were recorded with a Horiba Jobin Yvon Fluoromax-4 or a Perkin-Elmer LS-55 or an Edinburgh FLS920 equipped with a photomultiplier Hamamatsu R928 phototube. The same instrument connected to a TCC900 card was used for Time Correlated Single Photon Counting (TCSPC) experiments with an LDH-P-C 405 or 635 pulsed diode laser. The fluorescence quantum yields (uncertainty, ± 15%) according to the standard method. [2] The emission intensities were corrected taking into consideration the inner filter effect. [2] For the fluorescence anisotropy measurements an Edinburgh FLS920 equipped with Glan-Thompson polarizers was used. The data were corrected for polarization errors using the G-factor.

*1.3.1 Fitting of the fluorescence decay to determine the excited state lifetimes*

In order to determine the excited state lifetime, the TCSPC decays were fitted either with a mono-exponential model:

$$I(t) = A + Be^{-\frac{t}{\tau}}$$

Or with a bi-exponential model:

$$I(t) = A + B_1e^{-\frac{t}{\tau_1}} + B_2e^{-\frac{t}{\tau_2}}$$

The analysis was performed using the software package FAST by Edinburgh Instruments.

The experimental traces, the fitted curves and the residuals for RM and CM at 660 nm are shown in Figure S1.

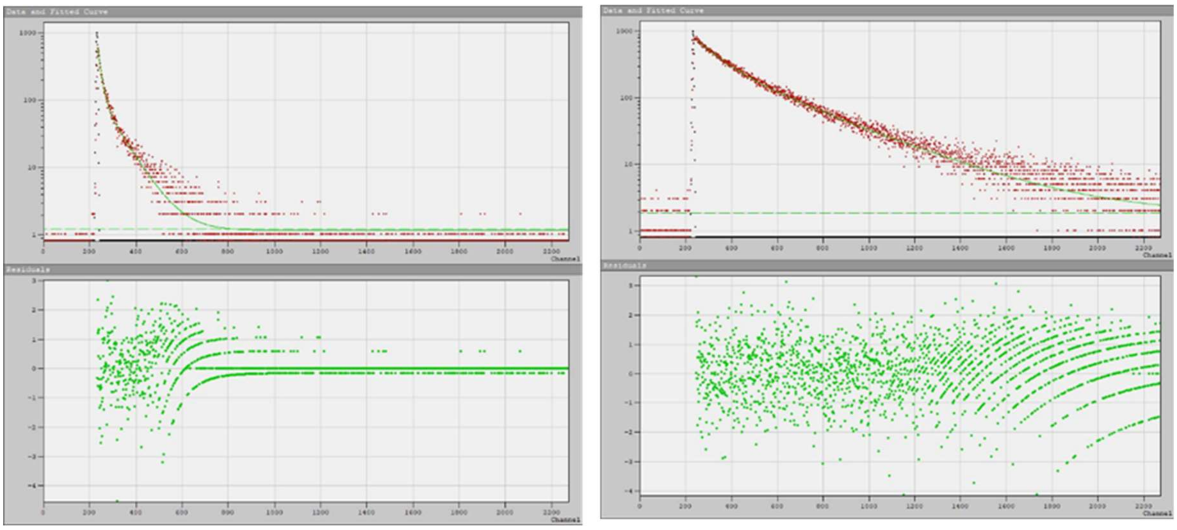

**Figure S1**. Experimental traces for RM and CM at 660nm as well as the fitting of the fluorescence decays and the residuals obtained using FAST software

The percentage was calculated as:

*1.3.2 Determination of the fluorescence quantum yield*

Quantum yield was calculated according to a standard method. In particular, the integral of the fluorescence spectrum of the x solution ($Area_x$) was compared with the one of a standard ($Area_S$):

$$\Phi_x = \Phi_s \frac{Area_x}{Area_s} \frac{A_s}{A_x} \frac{n_x}{n_s}$$

Where $A_s$ and $A_x$ are the absorbance of the standard and x at the excitation wavelength and $n_s$ and $n_x$ the refractive indexes of the two solutions.

*1.3.3 Determination of the fraction of free polymer*

The determination of the fraction of free polymer was based on the fluorescence intensity and in particular when two emitting species are simultaneously present in solution the measured fluorescence quantum yield is:

$$\Phi = (\varepsilon_m \chi_m \Phi_m + \varepsilon_a \chi_a \Phi_a)$$

Where $\varepsilon_m$and $\varepsilon_a$ are the molar absorption coefficient of the monomer and of the aggregate at the excitation wavelength, $\Phi_m$and $\Phi_a$the fluorescence quantum yield of the monomer and of the aggregate and $\chi_m$and $\chi_a$the molar fractions of the monomer and of the aggregated molecules.

In the absence of aggregation:

$$\Phi^0 = \varepsilon_m \Phi_m$$

Considered that $\Phi_a \sim 0$

$$\frac{\Phi}{\Phi^0} = \chi_m$$

### 1.4 ESI-MS

The Mass spectra were performed at the UC San Diego Mass Spectrometry Laboratory on an Agilent 6230 Accurate-Mass TOF-MS.

### 1.5 NMR Spectroscopy

$^1$H-NMR spectra were recorded on a Varian MR400 instrument at room temperature. Chemical shifts are reported relative to the residual proton signal of the deuterated solvents.

### 1.6 Confocal Fluorescence Microscopy

Fluorescence microscopy images were acquired using a Leica SP5 II laser scanning confocal microscope. The 488, 555 and 405 lasers were used, respectively, to visualize micelles (perylene diimide), cell lysosomes (Lysotracker red) and cell nuclei (Hoechst).

### 1.7 Dynamic Light Scattering (DLS)

DLS measurements were performed with Zetasizer Nano ZS Malvern Panalytical using PMMA semi-micro cuvettes (BRAND).

### 1.8 Size Exclusion Chromatography (SEC-MALS)

Polymer molecular weights and polydispersity were determined by size-exclusion chromatography (Phenomenex Phenogel 5μm, $10^3$Å, 1K-75K, 300 x 7.8 mm in series with a Phenomenex Phenogel 5μm, $10^3$Å, 10K-100K, 300 x 7.8 mm) at 65 °C in 0.05 M LiBr in DMF, using a ChromTech Series 1500 pump equipped with a multi-angle light scattering detector (DAWN-HELIOS II, Wyatt Technology) and a refractive index detector (Wyatt Optilab T-rEX).

### 1.9 Transmission Electron Microscopy (TEM)

Dry state TEM was performed using a Hitachi HD-2300 STEM at 200 kV. Grids were glow-discharged for 30 seconds in a Pelco easiGlow glow-discharger at 15 mA with a chamber pressure of 0.24 mBar and then prepared by drop-casting 4 μL nanoparticle solutions followed by staining using a 2 % uranyl acetate solution. For cryo-TEM, 200-mesh Cu grids with a lacey carbon membrane were glow-discharged for 30 seconds in a Pelco easiGlow glow-discharger at 15 mA with a chamber pressure of 0.24 mBar. 4μL of the sample was then pipetted onto the grid and plunge-frozen into liquid ethane with an FEI Vitrobot Mark III cryo plunge freezing robot for 5 seconds with a blot offset of 0.5 mm. Grids were stored in liquid nitrogen until loaded into a Gatan 626.6 cryo transfer holder cooled down to -172C. Microscopy was performed with a Hitachi HT7700 tungsten emission TEM at 100kV, and data were collected on a Gatan Orius 2k x 4.67k digital camera.

### 1.10 Flow Cytometry

Flow cytometric analyses were performed on HeLa cells by using CytoFLEX S (Beckam Coulter). Fluorescent events were measured in the FITC channel (excitation 488 nm, emission filter 525/40 nm) and at least 10,000 events were recorded for each sample. Data analysis was performed with CytExpert (Beckam Coulter) and FlowJo™.

## 2. Synthesis and Characterization

### 2.1 Synthesis and characterization of monomer m1

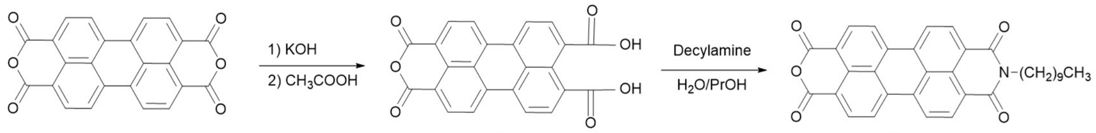


**Scheme S1.** Scheme of reaction mechanism for the synthesis of N-Decylperylene-3,4,9,10-tetracarboxylic-3,4-anhydride-9,10-imide (1c).

The perylene diimide monomer (m1) was synthetized by the partial hydrolysis of Perylene-3,4,9,10-tetracarboxylic acid dianhydride (PTCDA).[3] Briefly, 1.96 g (5 mmol) of PTCDA (1a) were dissolved in a 5% solution of potassium hydroxide (KOH) (22.4 g, 20 mM) at 90 °C leading to a solution with pH 10.5. Subsequently, a 14 g of 10% acetic acid ($CH_3COOH$) solution was added dropwise to the previous mixture over 30min maintaining the temperature stable at 90 °C and the pH was found to be in the range of 4.5-5.0. The suspension was stirred for 1 hour at the same temperature. The precipitated Bordeaux-coloured potassium salt (perylene-3,4,9,10-tetracarboxylic acid mono-anhydride mono-potassium salt (1b)) was filtered at room temperature, washed with water and dried at 130 °C. The purified product was obtained with a reaction yield of 95 % (2.2 g). The MS data were collected by negative ion mode electrospray ionization mass spectroscopy and matched the ones reported bibliographically with a main peak at 531 m/z and a second at 670 m/z due to the starting PTCDA. [4] In the next step, N-Decylperylene-3,4,9,10-tetracarboxylic-3,4-anhydride-9,10-imide (1c) was synthetized according to the bibliography [5] as summarized in Scheme S1 by mixing 1.25 g (2.79 mmol) of the previously synthetized product 1b and a 4.4 molar ratio of decylamine (1.93 g ,12.3 mmol) in 12 ml of $H_2O$-PrOH (v/v) 1:1 solvent mixture. The reactants, were stirred at room temperature for 4 hours and then were heated at 90 °C for more 2 hours, keeping the reaction under magnetic stirring. In the next step, the reaction mixture was acidified with 10% hydrochloric acid (HCl) and the precipitate was filtered and washed with water to ensure the elimination of the unreacted amine. The residue was afterwards stirred in hot 10% KOH and 8% potassium chloride (KCl) was added in order to separate the precipitated potassium salt of 1c and the symmetrically substituted diimide from the soluble unreacted 1b. The precipitated solid was stirred into water and the insoluble, symmetrically substituted diimide was removed. The filtrate was then precipitated with 20% HCl. The precipitate was filtered, washed with water, and dried to give 1c with 70.4 % yield. The NMR and MS data matched those reported.[6]

Following N-octylamine norbornene (1d) was synthetized by mixing overnight the mono-Boc-1,8 diaminooctane (7.9 mmol) in 50ml toluene with cis- 5-norbornene-exo-2,3-dicarboxylic anhydride (8.69 mmol) under heating with a dean stark trap in place. The next day the reaction was cooled down to room temperature and was concentrated until dryness. The product was purified by flash chromatography (3:7= hexane: ethylacetate) and was obtained as a clear oil (yield 75 %). The $^{1}$H-NMR (400MHz, $Cl_3CD$-d) results are reported: δ (ppm):: δ (ppm): 1.25 (m, 1H), 1.30 (m, 12H) 1.50 (m, 1H), 2.78 (m, 2H), 3.14 (m, 2H), 3.21 (m, 2H), 3.79 (t, 2H), 6.34 (m, 2H).[7] ESI+ (m/z) found: ($[M + H]^+$) = 291.2; calculated: 290.2.

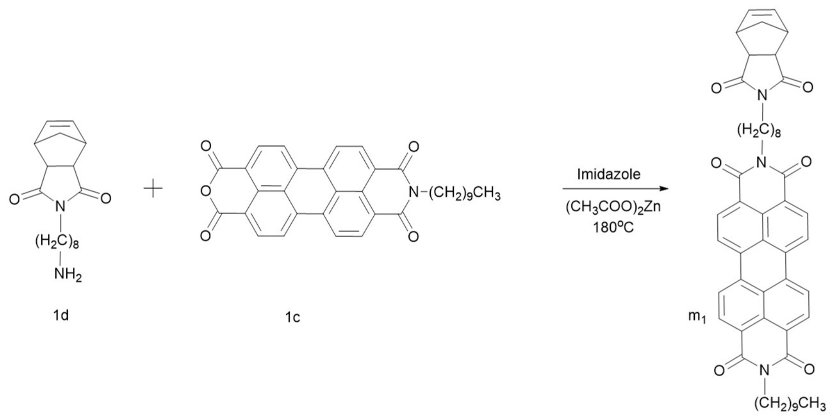


**Scheme S2**. Scheme of the reaction mechanism for the synthesis of N-Decyl,N'-octilnornbornin-3,4,9,10, perylene-dicarboxiimide (m1).

The final monomer N-Decyl,N'-Octilnornbornin-3,4,9,10, perylene-dicarboxiimide (m1) was synthesized by modifying an already existing protocol (Scheme S2).[8] Briefly, m1 resulted from the 4 hours condensation of 1d (1.00 mmol) and 1c (0.50 mmol) and imidazole (3.0 g) under stirring heated under $N_2$ at 180 °C in the presence of anhydrous zinc (II) acetate catalyst (0.44 mmol).[9] Also in this case flash chromatography was used as a purification technique in 9:1 DCM:MeOH with reaction yield of 75 %. The $^{1}$H-NMR (400MHz, $Cl_3CD$-d) results are reported: δ (ppm): 1.0-2.0 (broad, aliphatic), 2.66 (m, 2H), 3.25 (m, 2H), 3.50 (t, 2H), 4.1 (m, 4H), 6.25 (m, 2H), 8.50 (m, 4H aromatic), 8.65 (m, 4H aromatic). ESI- (m/z) found: ($[M - H]^-$) = 804.5; calculated: 803.4.

### 2.2 Synthesis and characterization of monomer m2

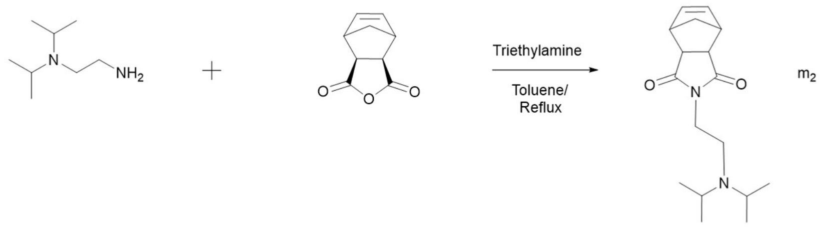


**Scheme S3.** Schematic synthesis of the tertiary amine norbornene derivative (m2).

The synthesis of the pH-responsive monomer (m2) was carried out as shown is Scheme S3. More in detail, m2 was synthetized by mixing 648 mg (4.5 mmol) *N,N*-diisopropylethylenediamine, 492 mg (3 mmol) cis-5-norbornene-exo-2,3-dicarboxylic anhydride and 606 mg (6 mmol) triethylamine in 50 ml dry toluene. The reaction was heated under reflux overnight. Then the solvent was evaporated under reduced pressure to minimum volume and the product was kept under vacuum 5 hours resulting in a brown oil (99% yield). The

$^1$H NMR (400MHz, $Cl_3CD$-d) results are reported: δ (ppm): 0.97 (m, 12H), 1.34 (m, 1H), 1.50 (m, 1H), 2.57 (m, 2H), 2.66 (m, 2H), 3.01 (t, 2H), 3.26 (m, 2H), 3.47 (t 2H), 6.27 (m, 2H). ESI+ (m/z) found: ($[M + H]^+$) = 291.2063; calculated: 291.2067.

**2.3 Synthesis and characterization of monomer m3**

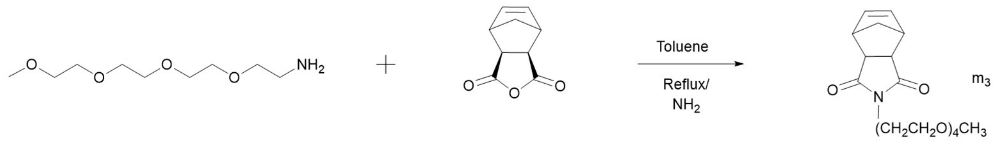


**Scheme S4.** Schematic synthesis of the hydrophilic monomer 2-(2,5,8,11-tetraoxatridecan-13-yl)-3a,4,7,7a-tetrahydro-1H-4,7-methanoisoindole-1,3(2H)-dione (m3).

The hydrophilic monomer 2-(2,5,8,11-tetraoxatridecan-13-yl)-3a,4,7,7a-tetrahydro-1H-4,7-methanoisoindole-1,3(2H)-dione (m3) was synthetized by heating in reflux overnight under nitrogen 1.5 g (9.1 mmol) cis-5-norbornene-exo-2,3-dicarboxylic anhydride with 2.27 g (11.0 mmol) 2,5,8,11-tetraoxatridecan-13-amine in 50 ml toluene according to Scheme S4. The reaction was cooled down to room temperature and then it was concentrated until dryness and purified by flash chromatography (2% MeOH in $CH_2Cl_2$) resulting in a light-yellow oil (97 % yield). The $^1$H NMR (400MHz, $Cl_3CD$-d) are reported: δ (ppm)1.35 (m, 1H), 1.47 (m, 1H), 2.66 (m, 2H), 3.24 (m, 2H), 3.36 (s, 3H), 3.5-3.7 (m, 16H), 6.26 (m, 2H). ESI+ (m/z) found: $[M + Na]^+$ = 376.1736; calculated. = 376.1730 .[10]

**2.4 Synthesis and characterization of monomer m4**

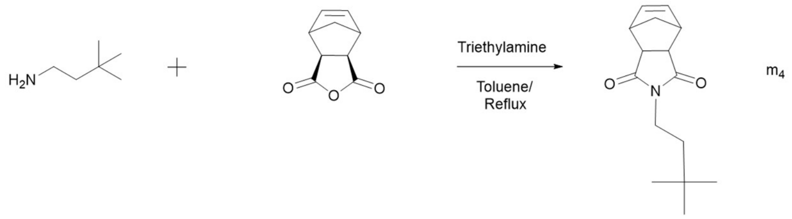


**Scheme S5.** Schematic synthesis of the non-pH-responsive monomer m4.

Monomer m4 was synthesized by reacting 368.8 mg (2.25 mmol) cis-5-norbornene-exo-2,3-dicarboxylic anhydride together with 334 µL (2.5 mmol) 3,3-Dimethylbutylamine and 620 µL (4.5 mmol) triethylamine in 10 mL dry toluene under reflux overnight (Scheme S5). The product was concentrated, dissolved in ethyl acetate and washed with water 3 times. The organic phase was dried over $MgSO_4$ and the solvent was evaporated to give the product (88 % yield) as a white solid. $^1$H NMR (400 MHz, $Cl_3CD$-d): δ (ppm) 0.97 (d, 9H), 1.22 (m, 1H), 1.42 (m, 2H), 1.50 (m, 1H), 2.65 (m, 2H), 3.26 (m, 2H), 3.44 (t, 2H), 6.27 (m, 2H).

## 2.5 Polymers R and C

.

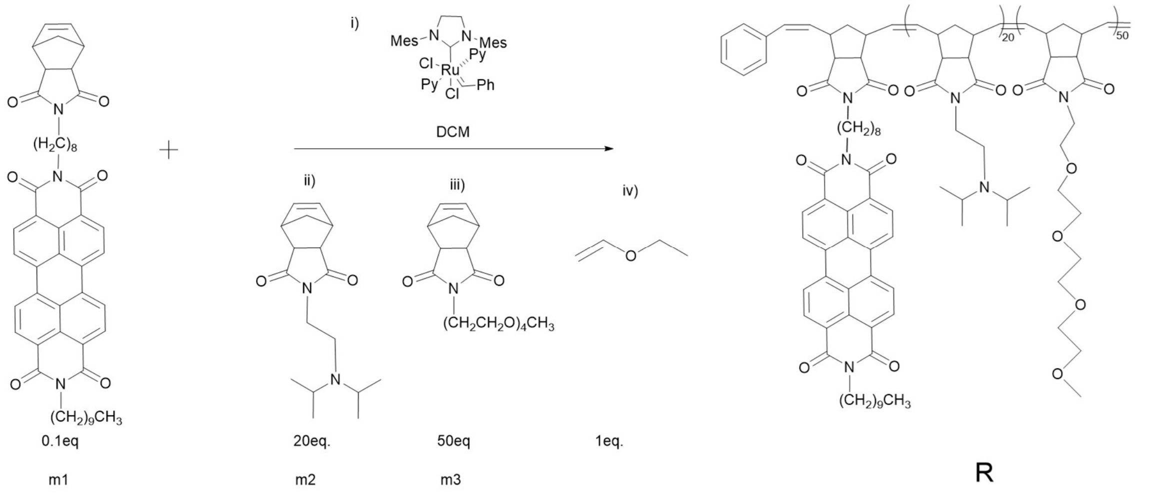


**Scheme S6**. Schematic synthesis of the pH-responsive polymer **R**.

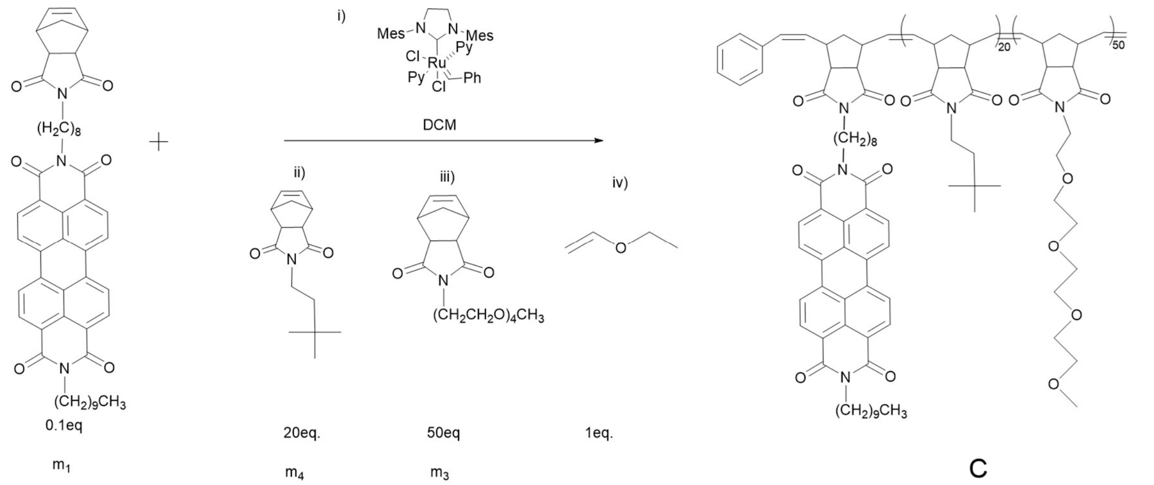


**Scheme S7**. Schematic synthesis of the control polymer **C**.

For the synthesis of the amphiphilic block copolymers (**R**, as an example) a Second-generation Grubbs catalyst ((IMesH2)(C5H5N)2(Cl)2Ru=CHPh) was exploited. As summarized in Scheme S.6, for the synthesis of R polymer 0.16 g of m1 (0.1:1 molar equivalent to the catalyst) were mixed in 400 µl dry dichloromethane (DCM) inside a dry Schlenk flask with a stir bar under $N_2$. For the polymerization of the monomer, a solution composed of 2.47 mg catalyst in 300 µl dry DCM was rapidly added to the mixture via a syringe, having the

flask under low stirring at room temperature. After 20 minutes, a solution of 11.6 mg (20:1 molar eq. to catalyst) m2 in 400 µl dry DCM was added via syringe to the flask and the polymerization reaction was stirred for additional 30 minutes. Afterwards, a solution of 33.5 mg m3 in 363 µl DCM (50:1 molar eq. to catalyst) was added and the reaction was stirred for 20 more minutes. Finally, an excess of ethyl vinyl ether (EVE) was added to the mixture in order to terminate the polymerization reaction. The obtained polymer was purified by two precipitation using diethyl ether after dissolution in DMF. The precipitated polymer was dried under vacuum. The accomplished polymerization was first confirmed by $^{1}$H NMR (400 MHz, DMF-d) (Figures S2 and Figure S3). Both spectra show the typical broad backbone polymer peaks (5.5-6 ppm) and the disappearance of the norbornene CH=CH monomer peaks at 6-6.5 ppm. In a second step, SEC was used to characterize the polymers. Fractions of the different block copolymers were collected during the synthesis, terminated with EVE, and the size of the blocks was analysed by SEC-MALS in DMF (Figure S4 and Table S1). The same reaction conditions were applied for the synthesis of polymer **C**, where instead of m2 monomer, m4 was added in a 20:1 equivalent to the catalyst. (Scheme S.7)

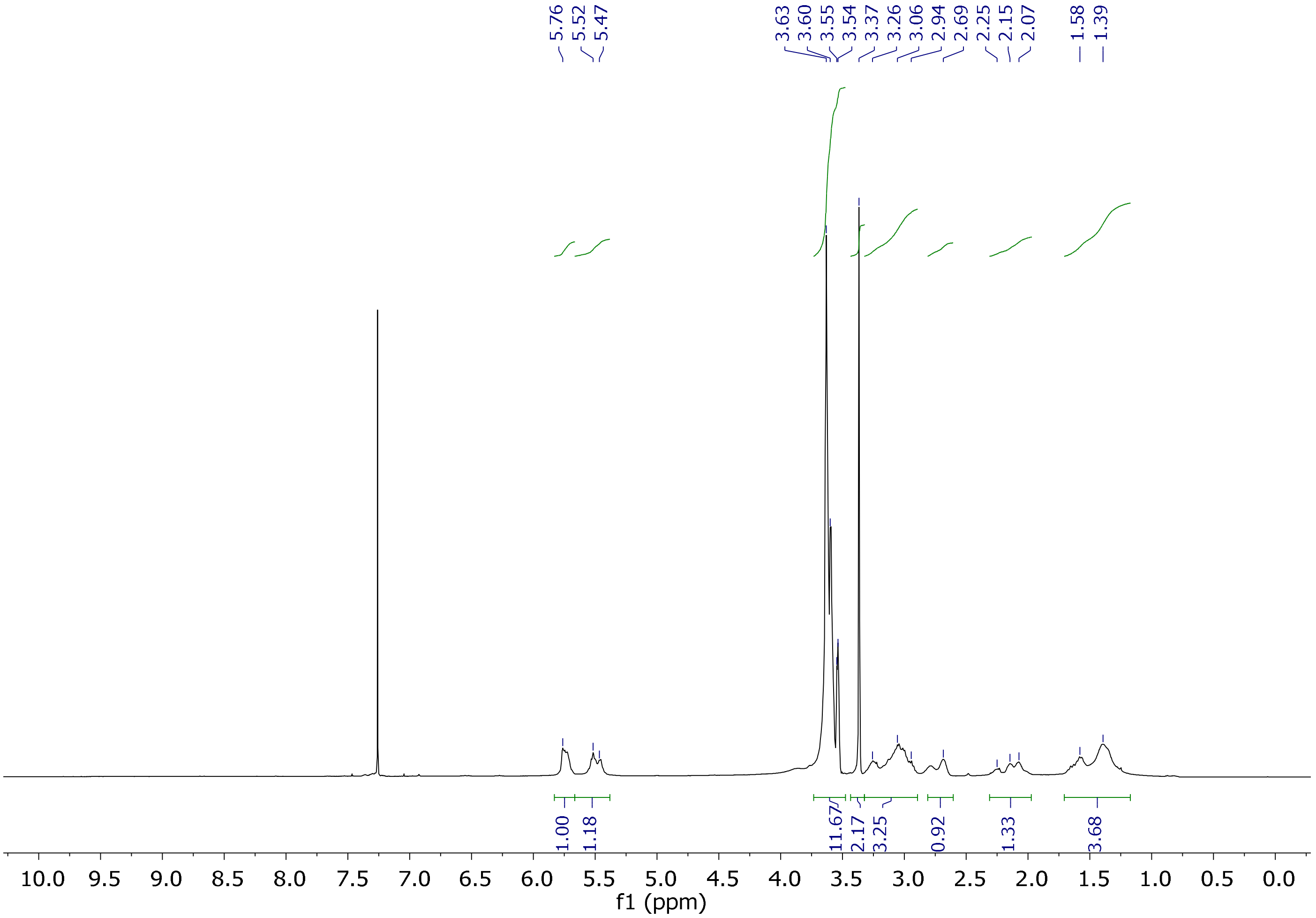


**Figure S2.** $^{1}$H-NMR (DMF-d) of polymer **R**.

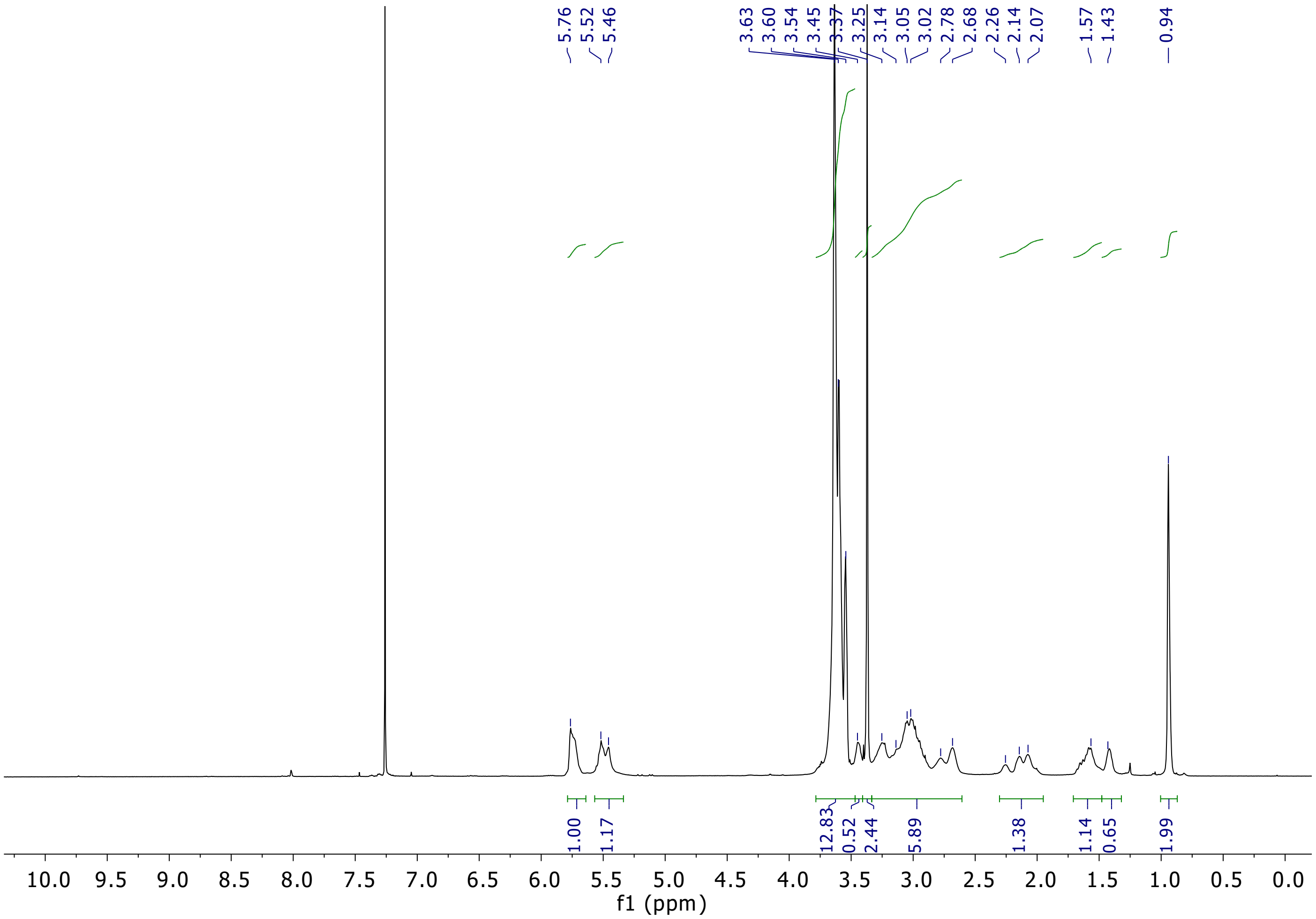


**Figure S3**. $^{1}$H-NMR (DMF-d) of polymer **C**.

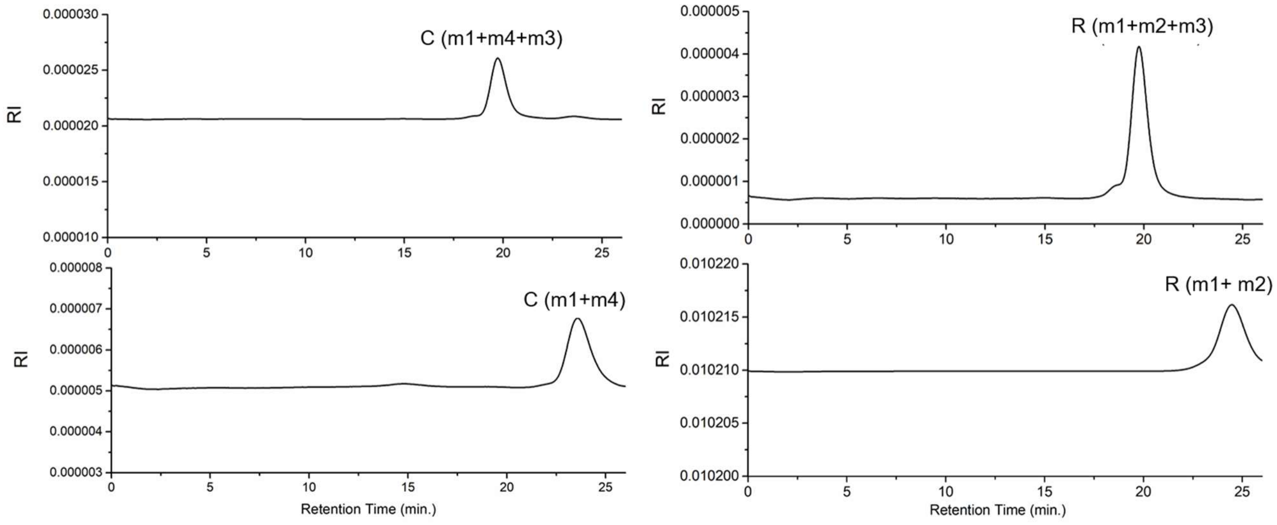


**Figure S4.** SEC-MALS of the block copolymers in DMF. Refractive index signal of the polymer blocks composed of monomer **m1** and **m4**/**m2** before the addition of **m3** (bottom) and of the final copolymers **R** and **C** (top).

| Copolymer | Mn (g/mol) | Polydispersity |
|---|---|---|
| **R (m1+m2+m3)** | 21060 | 1.069 |
| **C (m1+m4+m3)** | 18070 | 1.013 |

**Table S1.** Molecular weights and polydispersity of polymers **R** and **C** as determined by size exclusion chromatography.

## 2.6 Formation of RM and CM Micelles

For the formation of the nanoparticles the solvent switch method was adopted,[11] according to which a combination of a good and a bad solvent for the polymer is chosen. The polymers **R** and **C** were therefore, dissolved in DMF at a concentration of 2 mg/ml and were dialysed under gentle magnetic stirring against PBS 1X using 3,500 molecular weight cut off (MWCO) SnakeSkin dialysis tubing for 72 hours. The formation of the micelles was verified by DLS, where the formation of monodispersed micelles in the 15-30 nm hydrodynamic diameter range was found.

TEM was used to investigate the morphology of the nanostructures. For the TEM images, the sample was treated with 2 % uranyl acetate and deposited on a copper grid cover by Formvar. The grid was previously treated with low energy plasma, in order to clean the surface from environmental contamination and increase the hydrophilicity. TEM showed the presence of spherical micelles highly contrasted with respect to the background (Figure S5). 2% uranyl acetate staining was used to enhance contrast. These values were in accordance with DLS measurements.

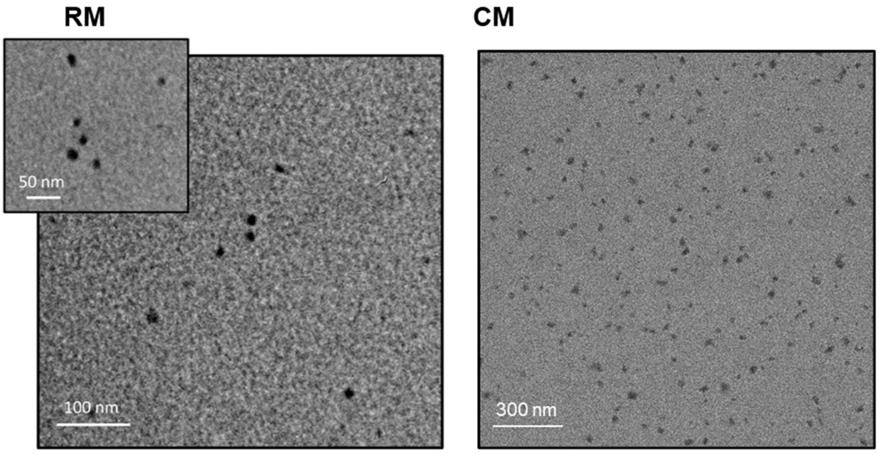


**Figure S5**: Dry state TEM images of the **RM** and **CM** in PBS stained with uranyl acetate, confirming the formation of spherical micelles at pH 7.4.

## 3. pH-responsive disaggregation of RM

To 100 µl of the **RM**/**CM** solution (2 mg/ml) 0, 5, 10, 20, 30, 40, 50 µl HCl 1 M were added gradually. The emission spectra were recorded after each addition by exciting the sample at λ = 470 nm, a wavelength where the monomer m1 containing the perylene group absorbs. A clear increase in the fluorescence intensity was observed upon a decrease of the pH verifying the disaggregation of the self-assembled nanoparticles followed by the "switch on" of the perylene diimide emission (Figure S6). The disaggregation of **RM** in acidic pH was also verified by DLS and compared to **CM** (Figure S7). Moreover, addition of the surfactant sodium dodecyl sulphate (SDS) was used to disassemble the pH insensitive **CM** micelles and compare their emission with that of the disassembled **RM** (Figure S6). Further investigation at pH 5.0 was carried out. Both **RM** and **CM** were dialysed for up to 48 hours in acetate buffer at pH = 5.0. The emission spectra were recorded after 2, 24 and 48 hours by exciting the sample at λ = 470 nm. The solutions were finally analysed by DLS (Figure S8) and Cryo-EM (Figure S9). Further imaging of **CM** via dry state TEM was performed to demonstrate the stability of **CM** at pH 5.0 (Figure S10).

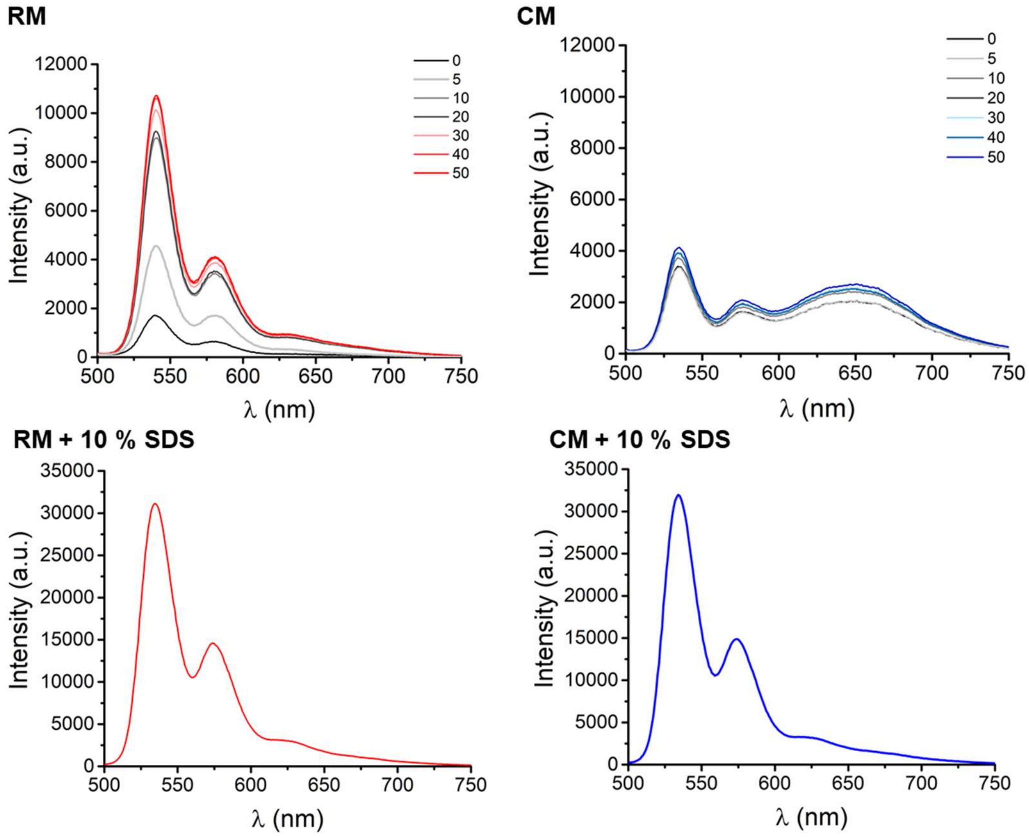


**Figure S6.** Fluorescence Intensity of **RM** and **CM** solutions before and after acidification. 0, 5, 10, 20, 30, 40, 50 µL HCl 1M were added to 100 µL micelle solutions (top panels). 10 % SDS was used to disassemble the remaining micelles (bottom panels).

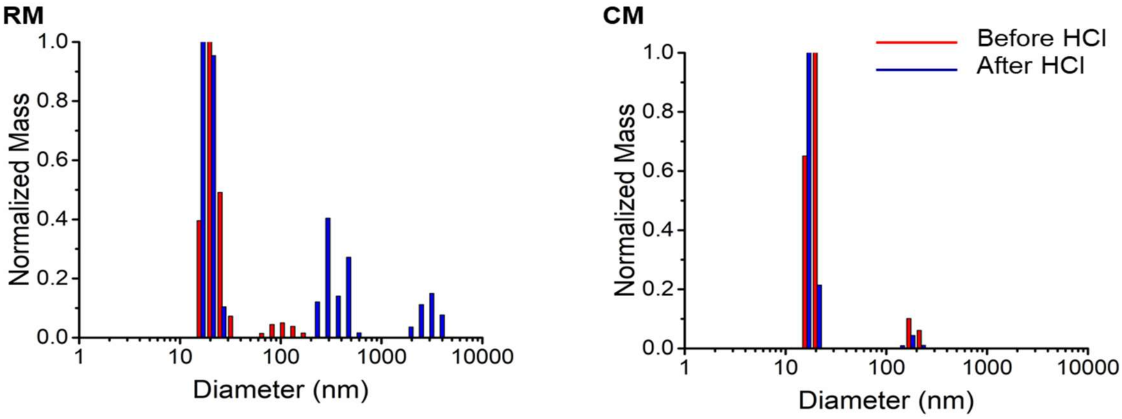


**Figure S7.** DLS of **RM** and **CM** before (red) and after (blue) treatment with HCl.

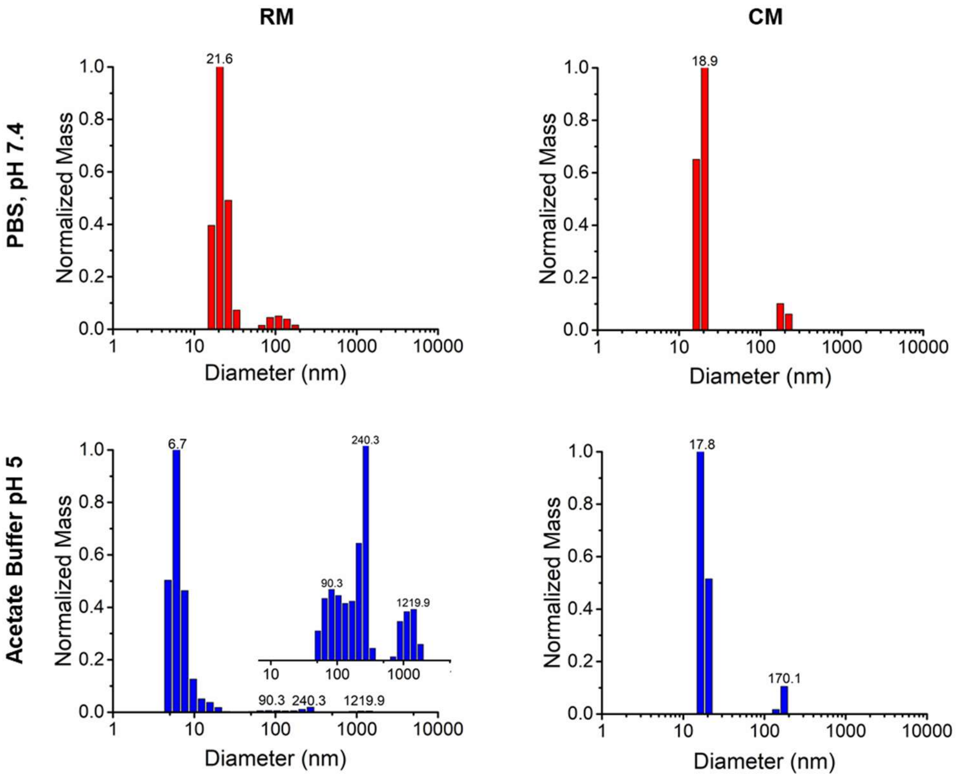


**Figure S8.** DLS of **RM** and **CM** in PBS pH=7.4 and after 48 h dialysis in acetate buffer pH =5.0.

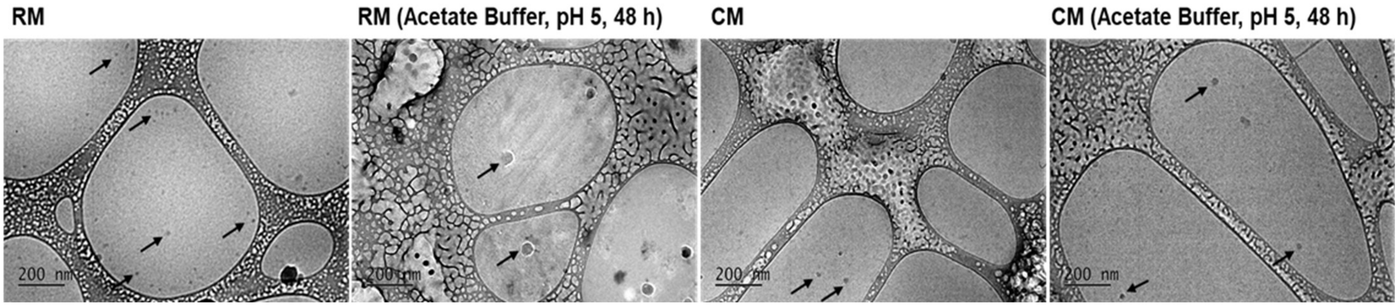

**Figure S9.** Cryo-TEM images of **RM** and **CM** before and after dialysis in acetate buffer at pH 5.0 for 48h.

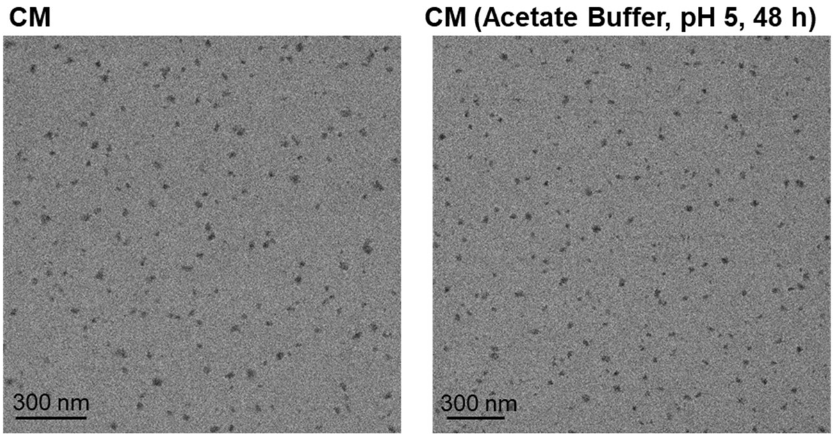


**Figure S10.** Dry state TEM images of **CM** before (left panel) and after (right panel) dialysis in acetate buffer at pH 5.0 for 48 h confirming the stability of **CM** at both pH 7.4 and pH 5.0. 2 % Uranyl acetate was used as staining.

## 4. Cell experiments

### 4.1 Flow Cytometry

Polymers R and C were diluted in complete DMEM (DMEM + FBS 10% + L-glutamine 2mM + PenStrep 1U/mL) at a final concentration of 200nM and were incubated for 2h, 24h or 48h together with 250,000 cells (HeLa). At least 10,000 events were analyzed on the CytExpert (Beckam Coulter) flow cytometer for their fluorescence in the FITC channel (excitation 488 nm, emission filter 525/40 nm). Events recorder and gating strategies applied on FSC/SSC dot plot are represented in Figure S11. An increase in fluorescence events as a function of incubation time is reported for both **RM** and **CM**. A significant increase in the fluorescence events for samples incubated with **RM** was observed after 24hours, while only a minor amount of the fluorescent events was observed in samples incubated with **CM**. The maximum number of fluorescent events was observed after 48h, with 42.54% fluorescence events for samples incubated with **RM**, compared to 5.23% of those incubated with **CM**.

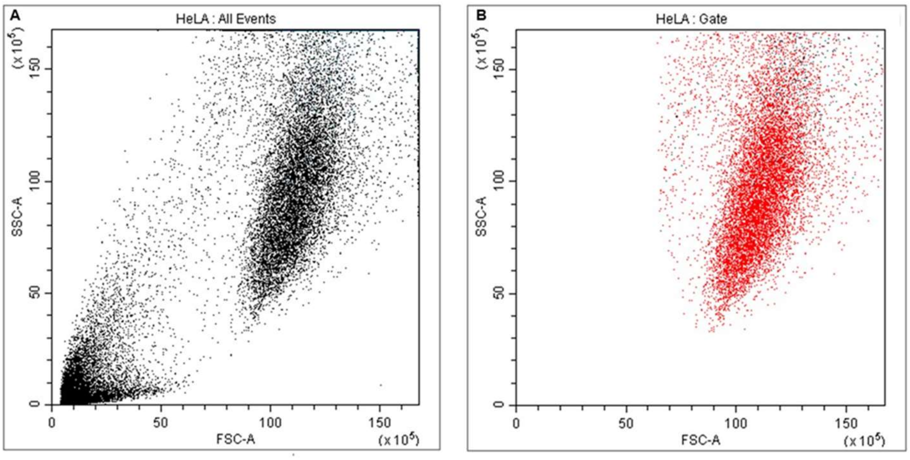


**Figure S11.** Flow-cytometric analysis of HeLa cells after incubation with either **RM** or **CM**. A) Representative gating plot of side scattering (SSC-A) versus forward scattering (FCS-A) of cells with all acquired events and B) gated on live cells.

### 4.2 Cytotoxicity

Compounds **RM** and **CM** were diluted in complete DMEM (DMEM + FBS 10% + L-glutamine 2mM + PenStrep 1U/mL) to final concentrations of 200nM, 20nM, 2nM,0.2nM and were incubated for 2h, 24h or 48h together with 20,000 cells (HeLa) (Figure S12). Cell viability was analyzed by MTT assay and the results were normalized on the untreated sample. According to the results, cytotoxicity was not observed in any of the analyzed conditions, therefore indicating **RM** and **CM** biocompatibility.

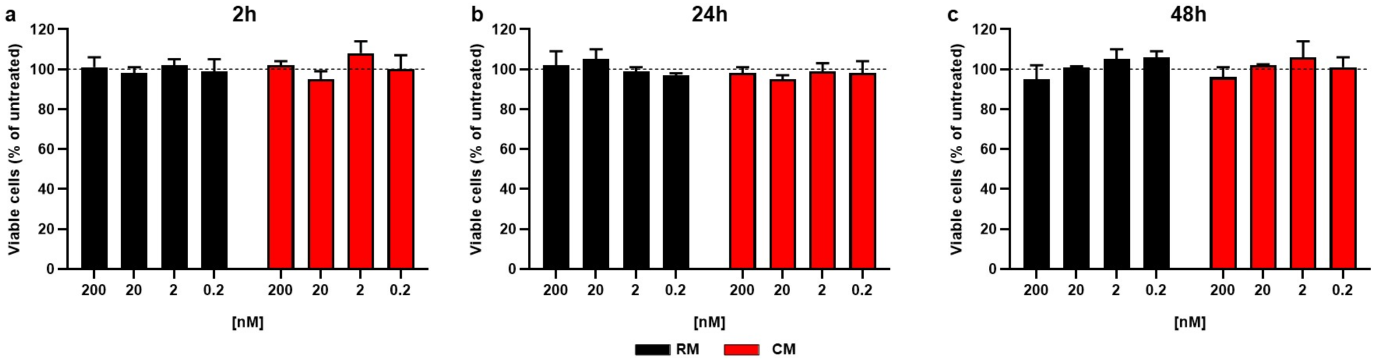


**Figure S12:** Cell viability after 2-, 24- and 48-hours incubations of HeLa cells with 200, 20, 2 and 0.2 nM of **RM** and **CM.**

### 4.3 Confocal Fluorescence microscopy

HeLa cells were maintained at 37 ℃ with 5 % $CO_2$ humidified atmosphere. Cells were kept in 25 $cm^2$ culture flasks in 5 mL DMEM supplemented with 10 % fetal bovine serum (FBS) and 1% penicillin/streptomycin. In a typical experiment, cells (150 μL) were seeded on a 4-well glass-bottom chamber at a density of 100,000 cells per well and incubated in medium for 24 h. After that, the medium was removed and 400 mL of either **RM** or **CM** solutions were added to the cells. Cells were incubated for 3 hours at 37 ℃, washed and incubated in medium without phenol red for additional 2, 24 or 48 h. Cell lysosomes were stained with lysotracker red and cell nuclei were stained with Hoechst (blue). All settings were kept constant for the acquisition of the fluorescence images (Figure S13).

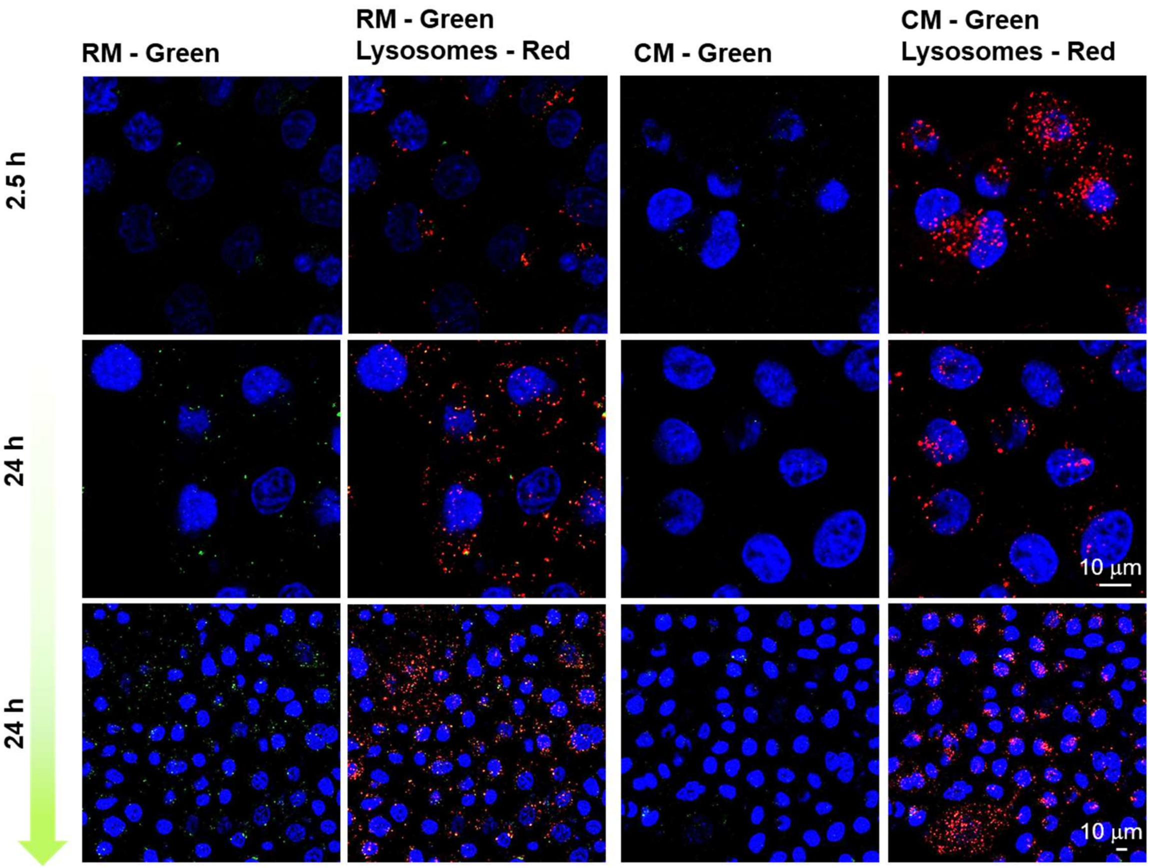


**Figure S13**. Confocal fluorescence microscopy images of HeLa cells incubated with either **RM** or **CM** for 3 h and post-incubated for either 2.5 or 24 h. The 24 h time point is presented in two different magnifications. Disassembled micelles are visible in the green channel and lysosomal vesicles were stained with lysotracker red. Cell nuclei were stained with Hoechst (blue).

### 4.4 Colocalization Analysis

Colocalization in fluorescence microscopy imaging can be described as the existence of two different fluorescence signals at the same pixel location and it presents results as a simple overlay made by different channels. Colocalization analysis strongly depends on the resolution, therefore confocal imaging is

appropriate for this type of analysis since it provides high resolution eliminating out-of-focus light.[12] Since the channels are generated by two different fluorophores, which in this case are lysotracker red and the disassembled **RM** or **CM** (green fluorescence), colocalization analysis of the confocal fluorescence microscopy images of HeLa cells was performed using ImageJ software. In particular, a JACoP plugin (Just Another Co-localization Plugin) was exploited, which evaluates the colocalization between two images and provides the Mander's overlap coefficients ($M_1$ and $M_2$) which give an indication of the proportion of the green signal concurring with a signal in the red channel over its total intensity. $M_1$ is the ratio of the sum of the intensities of pixels from the green image for which the intensity in the red channel is above zero to the total intensity in the green channel ($M_2$ reversed for red). Briefly, for each single incubation time (time zero, 2.5 hours and 24 hours) the three-color channels (RED, GREEN, BLUE) were split for each image and the red channel of the lysotracker was merged with the green channel of **RM** or **CM** resulting in a third image where the channels are combined and the overlapping pixels appear white. The colocalization images were finally combined with the blue channel for the nucleus. The values of the coefficients $M_1$ and $M_2$ are summarized in Table S2. As an example, for **RM** after 24 hours $M_1$ was found to be 0.258, while $M_2$ was found to be 0.885 which means that 26% of the red pixels colocalized with the green pixels while 89% of the green pixels colocalized with the red. On the contrary, for **CM** $M_1$ was less than 2% due to the fact that no disaggregation occurred to produce green emission.

The calculation of the Mander's overlap coefficient is based on the Pearson's correlation coefficient which can be calculated by the following equation:

$$r_P = \frac{\sum_i((A_i - a) \times (B_i - b))}{\sum_i(A_i - a)^2 \times \sum_i(B_i - b)^2}$$

Where channel $A$ and $B$ grey values of voxel i are noted as $A_i$ & $B_i$ respectively and their corresponding average intensities as $a$ and $b$.

By using the same equation without subtracting the mean intensity values, the overlap coefficient, $r$, is calculated:

$$r = \frac{\sum_i((A_i \times B_i)}{\sum_i(A_i - a)^2 \times \sum_i(B_i - b)^2}$$

$k1$ and $k2$ coefficients are two components of the overlap coefficient that are related to the total intensity of the two channels:

$$r^2 = k_1 \times k_2$$

$$k_1 = \frac{\sum_i(A_i \times B_i)}{(\sum_i(A_i)^2)} \quad k_2 = \frac{\sum_i(A_i \times B_i)}{(\sum_i(B_i)^2)}$$

Finally, the Mander's coefficients, $M_1$ & $M_2$, are calculated by:

$$M_1 = \frac{\sum_i (A_{i,coloc})}{\sum_i (A_i)} \qquad M_2 = \frac{\sum_i (B_{i,coloc})}{\sum_i (B_i)}$$

With $A_{i,colco}$ being $A_i$ if $B_i > 0$ and 0 if $B_i = 0$, while $B_{i,coloc}$ being $B_i$ if $A_i > 0$ and 0 if $A_i = 0$.[13,14]

| Sample | $M_1$ | $M_2$ |
|---|---|---|
| **RM** time zero | $M_1$=0.070 | $M_2$=0.978 |
| **RM** 2.5 hours | $M_1$=0.061 | $M_2$=0.774 |
| **RM** 24 hours | $M_1$=0.258 | $M_2$=0.885 |
| **CM** time zero | $M_1$=0.012 | $M_2$=1.000 |
| **CM** 2.5 hours | $M_1$=0.022 | $M_2$=0.614 |
| **CM** 24 hours | $M_1$=0.028 | $M_2$=0.648 |

**Table S2:** Mander's coefficient $M_1$ and $M_2$ estimated from the confocal fluorescence microscopy images of HeLa cells after incubation with **RM** and **CM** for 2.5 and 24 hours using ImageJ software with JACoP plugin. A corresponds to the red channel while B to the green channel, while $M_1$ represents the fraction of A overlapping B and $M_2$ the fraction of B overlapping A.

## 5. References


1. Nechmad, N.; Lemcoff, N., Sulfur-Chelated Ruthenium Olefin Metathesis Catalysts. *Synlett* **2020,** *24*.
2. Montalti, M.; Credi, A.; Prodi, L.; Gandolfi, M. T.; Michl, J.; Balzani, V., *Handbook of photochemistry*. 2020.
3. Iverson, I. K.; Tam-Chang, S.-W., Cascade of Molecular Order by Sequential Self-Organization, Induced Orientation, and Order Transfer Processes. *Journal of the American Chemical Society* **1999,** *121* (24), 5801-5802.
4. Tröster, H., Untersuchungen zur Protonierung von Perylen-3,4,9,10-tetracarbonsäurealkalisalzen. *Dyes and Pigments* **1983,** *4* (3), 171-177.
5. Yang, X.; Xu, X.; Ji, H.-F., Solvent Effect on the Self-Assembled Structure of an Amphiphilic Perylene Diimide Derivative. *The Journal of Physical Chemistry B* **2008,** *112* (24), 7196-7202.
6. Nagao, Y., Synthesis and properties of perylene pigments. *Progress in Organic Coatings* **1997,** *31* (1), 43-49.
7. Thompson, M. P.; Randolph, L. M.; James, C. R.; Davalos, A. N.; Hahn, M. E.; Gianneschi, N. C., Labelling polymers and micellar nanoparticles via initiation, propagation and termination with ROMP. *Polymer Chemistry* **2014,** *5* (6), 1954-1964.
8. Huang, C.; Sartin, M. M.; Siegel, N.; Cozzuol, M.; Zhang, Y.; Hales, J. M.; Barlow, S.; Perry, J. W.; Marder, S. R., Photo-induced charge transfer and nonlinear absorption in dyads composed of a two-photon-absorbing donor and a perylene diimide acceptor. *Journal of Materials Chemistry* **2011,** *21* (40), 16119-16128.

9. Würthner, F., Perylene bisimide dyes as versatile building blocks for functional supramolecular architectures. *Chemical Communications* **2004,** (14), 1564-1579.
10. Hahn, M. E.; Randolph, L. M.; Adamiak, L.; Thompson, M. P.; Gianneschi, N. C., Polymerization of a peptide-based enzyme substrate. *Chemical Communications* **2013,** *49* (28), 2873-2875.
11. Barnhill, S. A.; Bell, N. C.; Patterson, J. P.; Olds, D. P.; Gianneschi, N. C., Phase Diagrams of Polynorbornene Amphiphilic Block Copolymers in Solution. *Macromolecules* **2015,** *48* (4), 1152-1161.

12. Zinchuk, V.; Zinchuk, O.; Okada, T., Quantitative colocalization analysis of multicolor confocal immunofluorescence microscopy images: Pushing pixels to explore biological phenomena. *Acta Histochemica et Cytochemica* **2007**, 40, 101–111.

13. Manders, E.M.M.; Stap, J.; Brakenhoff, G.J.; Van Driel, R.; Aten, J.A., Dynamics of three-dimensional replication patterns during the S-phase, analysed by double labelling of DNA and confocal microscopy. *Journal of Cell Science* **1992,** 103, 857–862.

14. Costes, S. V.; Daelemans, D.; Cho, E.H.; Dobbin, Z.; Pavlakis, G.; Lockett, S., Automatic and quantitative measurement of protein-protein colocalization in live cells. *Biophysical Journal* **2004**, 86, 3993–4003, doi:10.1529/biophysj.103.038422.
